# Energy Availability Determines Strategy of Microbial Amino Acid Synthesis in Volatile Fatty Acid–Fed Anaerobic Methanogenic Chemostats

**DOI:** 10.3389/fmicb.2021.744834

**Published:** 2021-10-04

**Authors:** Jian Yao, Yan Zeng, Miaoxiao Wang, Yue-Qin Tang

**Affiliations:** College of Architecture and Environment, Sichuan University, Chengdu, China

**Keywords:** thermophilic methanogenic community, functional groups, available energy, amino acids synthesis strategy, amino acids exchange

## Abstract

In natural communities, microbes exchange a variety of metabolites (public goods) with each other, which drives the evolution of auxotroph and shapes interdependent patterns at community-level. However, factors that determine the strategy of public goods synthesis for a given community member still remains to be elucidated. In anaerobic methanogenic communities, energy availability of different community members is largely varied. We hypothesized that this uneven energy availability contributed to the heterogeneity of public goods synthesis ability among the members in these communities. We tested this hypothesis by analyzing the synthetic strategy of amino acids of the bacterial and archaeal members involved in four previously enriched anaerobic methanogenic communities residing in thermophilic chemostats. Our analyses indicate that most of the members in the communities did not possess ability to synthesize all the essential amino acids, suggesting they exchanged these essential public goods to establish interdependent patterns for survival. Importantly, we found that the amino acid synthesis ability of a functional group was largely determined by how much energy it could obtain from its metabolism in the given environmental condition. Moreover, members within a functional group also possessed different amino acid synthesis abilities, which are related to their features of energy metabolism. Our study reveals that energy availability is a key driver of microbial evolution in presence of metabolic specialization at community level and suggests the feasibility of managing anaerobic methanogenic communities for better performance through controlling the metabolic interactions involved.

## Introduction

In most natural environments, microbial individuals rarely live alone but co-colonize with other species to form complex communities. Members in these communities are connected through intricate interaction networks. These interactions not only influence the growth and survival of individual member, but also scale up to determine the assembly and functions of the whole community (Zengler and Zaramela, 2018). Among diverse modes of microbial interactions, exchange of public goods (PGs) among different members is one of the most pervasive (Mitri and Foster, 2013), in which one member secretes metabolites to environment that benefit other members in the community. In turn, this community member can also obtain production from others. Previous studies suggest that many biologically essential metabolites can be shared as PGs, such as amino acids (AAs) (Mee et al., 2014; Embree et al., 2015; Hubalek et al., 2017), vitamins (Croft et al., 2005; Rodionova et al., 2015), siderophores (Kramer et al., 2020), and other cofactors (Jones, 1967). Associated with PG sharing, several genomic investigations indicate that many microorganisms in diverse environments [such as methanogenic chemostats (Hubalek et al., 2017), oil reservoir (Liu et al., 2018), and human gut (Soto-Martin et al., 2020)] contain only a small set of genes that encode these public functions, so they must survive by exchanging PGs with other members. This phenomenon reflects that PG sharing is a main force to drive the microbial genomic evolution (Morris et al., 2011) and play important roles in governing the assembly of the community (Zengler and Zaramela, 2018). Nevertheless, studies also suggest that the retained public functions of these auxotrophies were highly diverse (Hubalek et al., 2017; Liu et al., 2018; Soto-Martin et al., 2020). Given a strain residing in a community, we still lack knowledge to explain why it possesses the ability to execute the observed set of public functions (Zengler and Zaramela, 2018). The cause of this Gordian knot is that whether autonomously producing a specific PG benefits the community member itself is determined by many unknown biotic and abiotic factors present in complex natural communities. Uncovering these factors is crucial for understanding microbial evolution at community scale, as well as uncovering the assembly rule of microbial community with complex interaction networks.

The Black Queen Hypothesis (BQH) formulated by Morris et al. (2012) emphasizes that whether retaining (or loss of) a specific public function is selectively favored is determined by the traits of this function, including its energy cost, its essentiality, and its leakiness. A number of studies validated this prediction through theoretical models (Oliveira et al., 2014; Estrela et al., 2016; Mas et al., 2016; Zomorrodi and Segrè, 2017; Pacheco et al., 2019; Meijer et al., 2020; Wang et al., 2021), as well as experiments using synthetic microbial communities composed of engineered microbial auxotrophies (Mee et al., 2014; Pande et al., 2014; Cooper et al., 2019). These studies indicate that a leaky and essential public function was easier to be retained if the energy cost of performing this function is lower. Several studies extended the BQH framework to explain the scenario when multiple public functions can be shared, suggesting that the costs of all the function would profoundly affect the strategies of the strains involved (Mee et al., 2014; Embree et al., 2015). However, these studies simply assumed that the community members possessed similar metabolic functions except for their specific public functions. In addition, many studies explored the PG exchange strategies among community members in complex natural communities, such as anaerobic hydrocarbon-degrading community (Embree et al., 2015), freshwater mixed culture (Garcia et al., 2015), and kefir microbial community (Blasche et al., 2021). These studies showed that complex PG exchange relationships between community members were important causes for the stable coexistence of diverse species. Unfortunately, the factors that drive to these complex modes of PG exchange have not been fully revealed.

In complex natural community, the fitness of different members are highly varied and affected by complex factors, such as ecological niche (Neis et al., 2015), energy availability (Zengler and Zaramela, 2018), temporal shifts in community composition (Merchant and Helmann, 2012), and spatial structure (Allen et al., 2013). The fitness variation may also influence the strategies of PG production of the members involved. For example, if a community member possesses an ecological niche that could gain more energy to support PG production, it may possess ability to perform more public functions, even for those costly ones, because producing these PGs may only consume a relative smaller set of energy accounting for the overall energy it could obtain. Extending the framework of BQH, this hypothesis can be formulated as follows:


(1) $$p\propto \frac{ea}{c}$$

This relationship depicts that, in a complex community, the probability of whether a member can perform a public function (*p*) is (1) negatively correlated with the energy cost of the function (*c*) and (2) positively correlated with the overall energy availability for a member (*ea*).

In this study, we set out to test this hypothesis in anaerobic methanogenic communities (AMCs). Compared with other communities, the available energy is relatively limited in the AMC system and thus plays fundamental roles in driving the evolution of the community members involved (Lyu et al., 2018), as well as being associated with the metabolic interactions among these members (Jackson and McInerney, 2002; Nobu et al., 2020). In our previous works, we enriched four AMCs in thermophilic methanogenic chemostats supplemented with acetate, propionate, butyrate, or isovalerate as sole carbon source (accordingly, the four chemostats were simplified as ATL, PTL, BTL, and VTL thereafter) (Zheng et al., 2019; Chen et al., 2020a,b). Because AA is one type of important public functions that have been reported in many microbial communities [e.g., in ocean (Barbara and Mitchell, 2003) and in human gut (Perna et al., 2019), as well as other AMC systems (Embree et al., 2015; Hubalek et al., 2017)], we analyzed the abilities of AA synthesis of syntrophic acetate-oxidizing bacteria (SAOBs) involved in our enriched AMCs and found that AA synthesis ability was deficient in most of the SAOBs in our AMCs (Zeng et al., 2021). However, SAOBs represent only a subset of community members in AMCs. The PG synthesis strategies of other groups, as well as the underlying determinants, still remain poorly understood.

**FIGURE 1:**
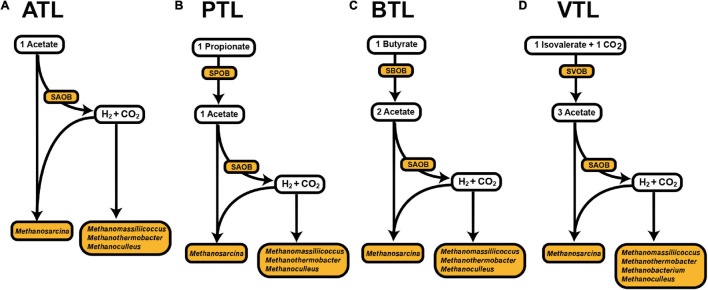
Proposed main carbon flow in methanogenic process in the four anaerobic chemostats (Chen et al., 2020a, b; Zheng et al., 2019). The ATL **(A)**, PTL **(B)**, BTL **(C)**, and VTL **(D)** represent the thermophilic methanogenic chemostats supplemented with acetate, propionate, butyrate, and isovalerate as sole carbon source, respectively.

Several functional groups dominated our four AMCs. In ATL, *Methanosarcina* can directly utilize acetate for methanogenesis through the acetotrophic pathway. In addition, the H_2_ and CO_2_ generated from acetate oxidation by SAOBs can also be utilized by *Methanosarcina*, *Methanomassiliicoccus*, *Methanothermobacter*, *Methanobacterium*, and *Methanoculleus* to produce methane (Supplementary Table 1). In PTL, one additional group of bacteria, syntrophic propionate-degrading bacteria (SPOB), is active to degrade propionate to acetate (Supplementary Table 1 and Figure 1B). Similarly, syntrophic butyrate-degrading bacteria (SBOB) degrade butyrate to acetate in BTL (Supplementary Table 1 and Figure 1C), whereas syntrophic isovalerate-degrading bacteria (SVOB) degrade isovalerate to acetate in VTL (Supplementary Table 1 and Figure 1D). In particular, microorganisms from different functional groups possess different energy availability, which is determined by the substrate availability (i.e., the fatty acids) in different environments, as well as their features of energy metabolisms. We thus expected to know whether the strategies of AA synthesis of the members in these functional groups are correlated with their energy availability, as well as the energy cost of the AA synthesis, which can be directly applied to test our hypothesis.

To this end, in this study, we analyzed the ability of AA synthesis of the communities in our methanogenic chemostats using our previously assembled metagenomic genomes, as well as the associated metatranscriptomic data.

## Materials and Methods

### Settings of the Chemostats Containing the Anaerobic Methanogenic Communities

In our previous work, four thermophilic (55°C) methanogenic chemostats (ATL, PTL, BTL, and VTL, fed with acetate, propionate, butyrate, and isovalerate as sole carbon sources, respectively) were built and operated at a low dilution rate (0.025 day^–1^, hydraulic retention time (HRT) = 40 days). The ATL and PTL were seeded with sludge from an anaerobic digester treating kitchen waste, whereas BTL and VTL were seeded with sludge from an anaerobic reactor treating swine manure. The total organic carbon (TOC) of synthetic wastewater fed to each chemostat was 8,000 mg L^–1^ (Supplementary Figure 1). These chemostats were stably operated over 200 days, and the biogas production was maintained at a constant level, and no fatty acids were accumulated in each chemostat. During the stable operation of these reactors, sludge samples were collected from ATL (days 306 and 307), PTL (day 223, 293, and 318), BTL (day 251 and 252), and VTL (day 295 and 296). The details about metagenomic and metatranscriptomic sample preparation, sequencing, assembly, and binning are available in the Supplementary Methods. More details about the operation and performance of chemostats are described in previous works (Chen et al., 2020a,b; Zeng et al., 2021).

### Analysis of Amino Acid Synthesis Strategy in the Extracted Metagenome-Assembled Genomes

Two hundred twenty-seven high-quality metagenome-assembled genomes (MAGs, contamination < 10%, completeness > 70%) were recovered in our previous study (see Supplementary Figure 2 for details about MAGs) (Chen et al., 2020a,b; Zeng et al., 2021), and raw sequence data were accessible at http://bigd.big.ac.cn/gsa (accession no. CRA004311). On the basis of these data, the gene sets related to AA synthesis were searched in these MAGs, by performing functional annotation on KEGG Automatic Annotation Server (Moriya et al., 2007). If an MAG had all the genes involved in the synthetic pathway of an AA (see Supplementary Figure 3 for the complete synthetic pathway of all the AAs), it was defined to possess the ability to synthesize this AA at genomic level, denoted by the blue box in Supplementary Figure 4.

Metatranscriptomic sequencing of these communities was also performed in our previous study (raw sequence data are accessible at http://bigd.big.ac.cn/gsa, accession no. CRA004311). RPKM-NM (Zheng et al., 2019; Chen et al., 2020a,b) values of all the genes involved in AA synthesis were extracted from our previous datasets (Supplementary Tables 2–5). To quantify the activity of synthesis of an AA in our MAGs at transcriptional level (*activity*_*a.a.*_), RPKM-NM values of all genes involved in its synthetic pathway were summed and divided by the number of genes, formulated as follows:


(2) $$Activity_{a.a.}=\frac{\sum_{1}^{n}RPKM-NM_{gene\;i}}{N\left(steps\right)}$$

In order to compare the overall AA synthesis activity among different MAGs, we defined the relative AA synthesis activity (*RA*_*a.a*_.) as follows:


(3) $$RA_{a.a.}=Activity_{a.a}\times \frac{MAG_{i}reads}{\sum_{1}^{n}MAG_{i}reads}$$

Here, *MAG_*i*_ reads* represents the total number of reads that can be mapped to metatranscriptomic data for all genes in the *i*th MAG.

We quantified the relative contribution of AA synthesis of each functional group by defining *Contribution*_*f.g.*_ based on *RA*_*a.a.*_ as follows:


(4) $$Contribution_{f.g.}=\frac{\sum_{1}^{j}RA_{a.a.}ofMAG_{i}}{\sum_{1}^{n}RA_{a.a.}ofMAG_{i}}$$

Here, *j* represents the number of MAGs within a functional group; *n* represents the number of all MAGs.

### Statistical Analysis

The significance analysis of the difference in AA synthesis ability among different functional groups is performed using the “stats” package (4.0.2) of R (4.0.2) through analysis of variance. Correlation analyses were performed using Pearson correlation method using the cor.test() function of the stats package (4.0.2) in R (4.0.2).

## Results

### Overall Strategy of Microbial Amino Acid Synthesis in the Four Anaerobic Methanogenic Communities

In our previous study, we recovered 57, 63, 52, and 55 high-quality MAGs from four enriched AMCs residing in ATL, PTL, BTL, and VTL, respectively. Nineteen MAGs were identified as methanogens at genus level, including four *Methanomassiliicoccus* (ATL89, PTL47, BTL15, and VTL23), four *Methanosarcina* (ATL14, PTL77, BTL39, and VTL53), one *Methanobacterium* (VTL28), six *Methanothermobacter* (ATL45, PTL2, PTL149, BTL70, VTL26, and VTL90), and four *Methanoculleus* (ATL103, PTL54, BTL76, and VTL77). Forty MAGs (out of 208 bacterial MAGs in total) were identified as potential SAOBs, because these MAGs contain genes encoding the WL or WL–glycine cleavage pathway, as well as complementary NADPH reoxidation and H2/formate-generating enzymes (Zeng et al., 2021). Similarly, one, two, and one MAGs were identified as SPOB, SBOB, and SVOB, respectively, including *Pelotomaculum* (PTL62) (Chen et al., 2020b), unclassified *Clostridiales* (VTL56) (Chen et al., 2020a), *Syntrophomonas* (BTL36) (McInerney et al., 1981), and *Syntrophothermus* (BTL6) (Luo et al., 2002). Meanwhile, we also found that many MAGs were highly abundant in the four AMCs, but did not contain the genes involved in the above core methanogenic pathways, which we defined as “noncore functional bacteria,” including 45 MAGs in ATL, 55 MAGs in PTL, 40 MAGs in BTL, and 42 MAGs in VTL. Notably, in each AMC, none of the community members contains all the genes responsible for the synthesis of 20 essential AAs (Supplementary Figure 4). The prevalent lack of AA synthesis capacity among MAGs suggested that metabolic interdependency relying on PG sharing is essential for the survival of these MAGs in our four AMCs.

**FIGURE 2:**
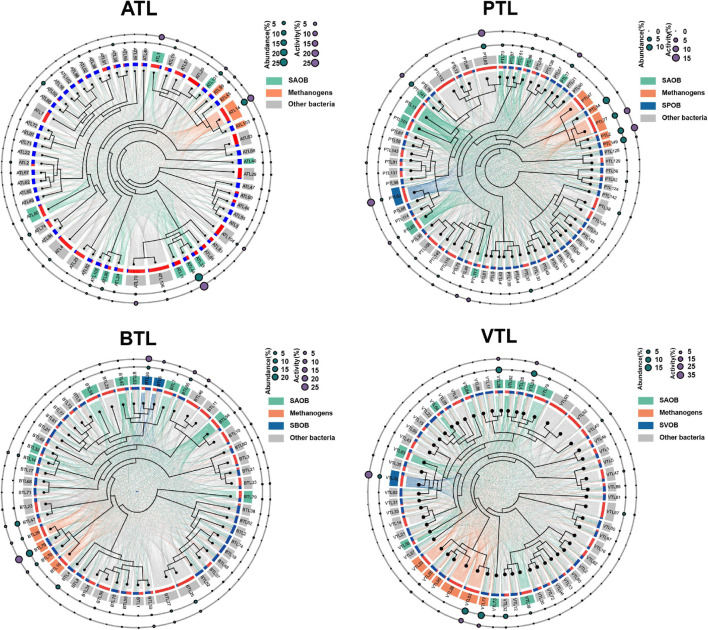
Potential complementarity in amino acid (AA) synthesis among the community members in each chemostat. The ATL, PTL, BTL, and VTL represent the thermophilic methanogenic chemostats supplemented with acetate, propionate, butyrate, and isovalerate as sole carbon source, respectively. The internal tree diagram depicts the phylogenetic relationship among the community members. The outer colored donuts indicate different functional groups, as shown in the left of each figure. All the members are connected by the lines reflecting their functional complementarity. Each link starts from an metagenome assembled genomes (MAG) that “contributes” AAs (labeled with the red patch) and ends with an MAG that “obtains” the AAs (labeled with the blue patch). The strength of the relationship is characterized by the thickness of the line. An additional diagram is given in Supplementary Figure 5. Accordingly, the width of red patch for a given MAG indicates its potential to “contribute” AAs to the community, whereas the width of blue patch for a given MAG indicates its potential to “cheat” AAs from the community. Dark green and purple bubbles in the outside concentric rings indicate the abundance and overall activity of community members, respectively.

In order to depict the complex interaction modes of AA sharing in our AMCs, we evaluated the potential complementarity in AA synthesis between every two MAGs in each AMC (Figure 2 and see Supplementary Methods for details about calculation). When an MAG contains all the genes encoding the enzymes used for the synthesis of an AA, it potentially “contributes” this AA to support the growth of other MAGs. In contrast, if an MAG does not contain these genes, they must “obtain” AAs produced by other MAGs. For each MAG, we counted how many AAs it could “contribute” to each MAG as the “contribute strength” (denoted as a link starting from the red patch in Figure 2). Similarly, we counted how many AAs it must “obtain” from one of the other MAGs as the “obtain strength” (denoted as a link ending in the blue patch in Figure 2). Then, the potential interactive connections between every pair of MAGs in each AMC were built. As shown in Figure 2, several MAGs tended to “contribute” more to others, whereas several MAGs “obtain” most of AAs from other members. To quantify this feature; we defined the contributing index (*ci*) of an MAG as the ratio of the summary of “contribute strength” to the summary of the “obtain strength” (see Supplementary Figure 5 for details). When the *ci* value (Supplementary Table 6) of an MAG was over 1, we defined it as a “contributor.” In contrast, MAGs with a *ci* less than 1 were defined as “beneficiaries.” We found that the number of “contributors” MAGs was lower than that of “beneficiaries” MAGs in every AMC (17 against 40 in ATL, 23 against 40 in PTL, 22 against 30 in BTL, and 21 against 34 in VTL), and the overall abundance of “contributors” was also lower than that of “beneficiaries” in ATL (32 < 59%), PTL (35 < 52%), BTL (37 < 59%), and VTL (42 < 49%). However, at transcriptional level, the total activity of “contributors” was higher than that of “beneficiaries” in ATL (50 > 42%), PTL (56 > 31%), and VTL (62 > 30%), except in BTL (27 < 71%). These results suggest that in AMCs, a minority of MAGs contribute to producing the majority of AAs to support the growth of the whole community, and these MAGs exhibit higher metabolic activities, which may facilitate the production of AAs. This phenomenon is very similar to the findings in other natural communities (Embree et al., 2015; Liu et al., 2018).

### Effect of Biosynthetic Cost on the Strategies of Amino Acid Synthesis

**FIGURE 3:**
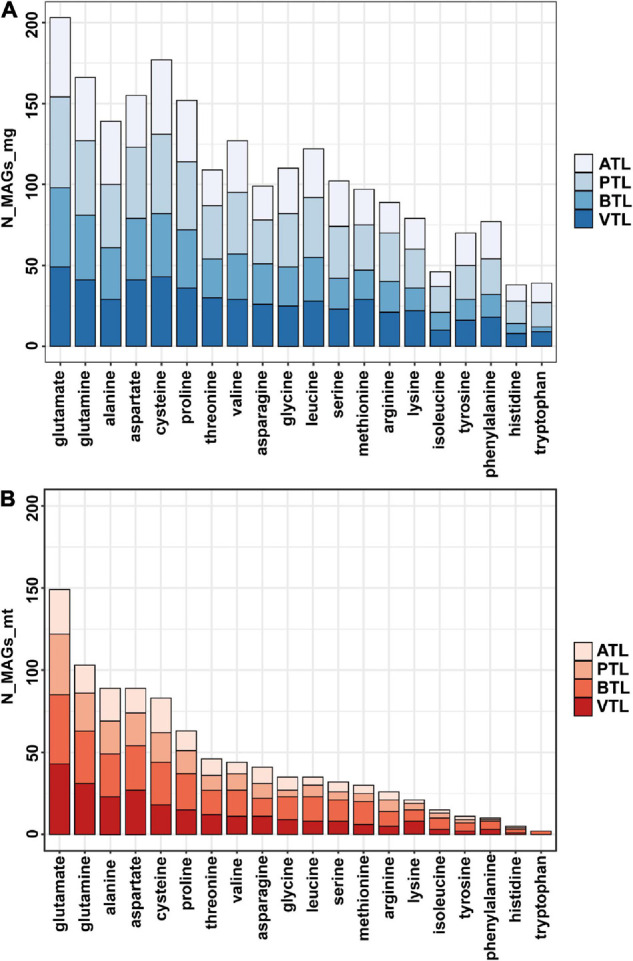
The total number of MAGs which can synthesize each amino acid in different chemostats at genome level **(A)** and transcriptome level **(B)**. The ATL, PTL, BTL, and VTL represent the thermophilic methanogenic chemostats supplemented with acetate, propionate, butyrate, and isovalerate as sole carbon source, respectively. “N_MAGs_mg” represents the number of MAGs that can synthesize each amino acid at genome level. “N_MAGs_mt” represents the number of MAGs that can synthesize each amino acid at transcriptomic level.

**FIGURE 4:**
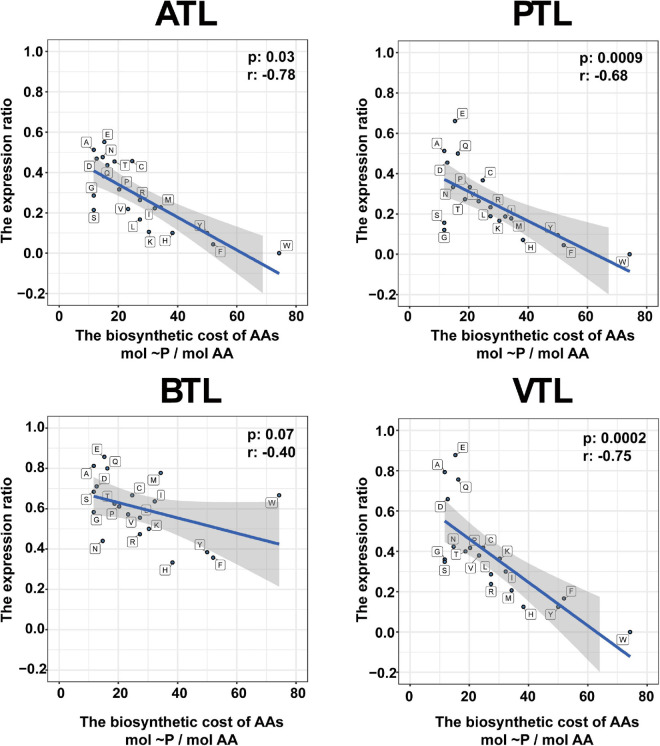
Relationship between the amino acid expression ratio and biosynthetic cost of each amino acid. The ATL, PTL, BTL, and VTL represent the thermophilic methanogenic chemostats supplemented with acetate, propionate, butyrate, and isovalerate as sole carbon source, respectively. The shaded area represents the 95% confidence region. The biosynthetic cost of each amino acid is represented by the number of high-energy phosphate bonds sacrificed in the biosynthetic pathway (Akashi and Gojobori, 2002). The *expression ratio* is defined as follows: $Expressionratio=\frac{N\left({\text{NAG}}_{\text{MT}}\right)}{N\left({\text{MAG}}_{\text{MG}}\right)}$. *N*(MAG_*MT*_) represents the number of MAGs in which the genes responsible for the synthesis of the corresponding AA were actively transcribed. *N*(MAG_*MG*_) is the number of MAGs that contain all the genes required for synthesizing the corresponding AA. Abbreviations of the amino acids: alanine (A), arginine (R), asparagine (N), aspartate (D), cysteine (C), glutamine (Q), glutamate (E), glycine (G), histidine (H), isoleucine (I), leucine (L), lysine (K), methionine (M), phenylalanine (F), proline (P), serine (S), threonine (T), tryptophan (W), tyrosine (Y), and valine (V).

Previous studies showed that, in natural communities, the frequency of members that could synthesize an AA is negatively correlated with its energy cost (Mee et al., 2014). Thus, we next tested whether this observation was also the case in our AMCs. To evaluate the energy cost of AAs, we followed the framework proposed by Akashi and Gojobori (2002), which assessed the net ATP consumption in the synthetic pathway of each AA. Our results indicated that, generally, the number of MAGs that synthesize the expensive AAs (e.g., histidine and tryptophan) was less than that of MAGs synthesize the inexpensive ones at genome level and transcriptome level (e.g., glutamate and glutamine; Figures 3A,B), consistent with the findings in previous studies. Statistically, the number of MAGs containing synthesis genes of an AA was negatively correlated with the function cost of the AA (Supplementary Figure 6A). Nevertheless, 77 and 70 MAGs possessed genes to synthesize phenylalanine and tyrosine, respectively, which was higher than the number of MAGs that possessed genes to synthesize relatively inexpensive histidine (38 MAGs) and isoleucine (46 MAGs) (Supplementary Figure 6A), seemly contradictory to the previously proposed rule. Interestingly, we found that the genes encoding AA synthesis in these 77 and 70 MAGs were lowly transcribed (Supplementary Figure 6B), suggesting that MAGs in our AMCs adapted a regulatory strategy to decrease the synthetic activity of these two costly AAs and thus saved energy for their better survival. Combining metagenomic and metatranscriptomic data, we defined an “expression ratio” to quantify the synthetic activity of synthesizing an AA in our communities (see legends to Figure 4 for details). Pearson correlation test showed a significant negative correlation between the expression ratio of AAs and their biosynthesis cost in ATL (*p* = 0.03, *r* = −0.78), PTL (*p* = 0.0009, *r* = −0.68), BTL (*p* = 0.07, *r* = −0.40), and VTL (*p* = 0.0002, *r* = −0.75; Figure 4). These results indicate that our AMCs have been optimized at transcriptional level to reduce the metabolic burden. Therefore, function cost is a key driver that affects the strategy of PG production of the MAGs in our AMCs.

### Strategies of Amino Acid Synthesis of Different Functional Groups

**FIGURE 5:**
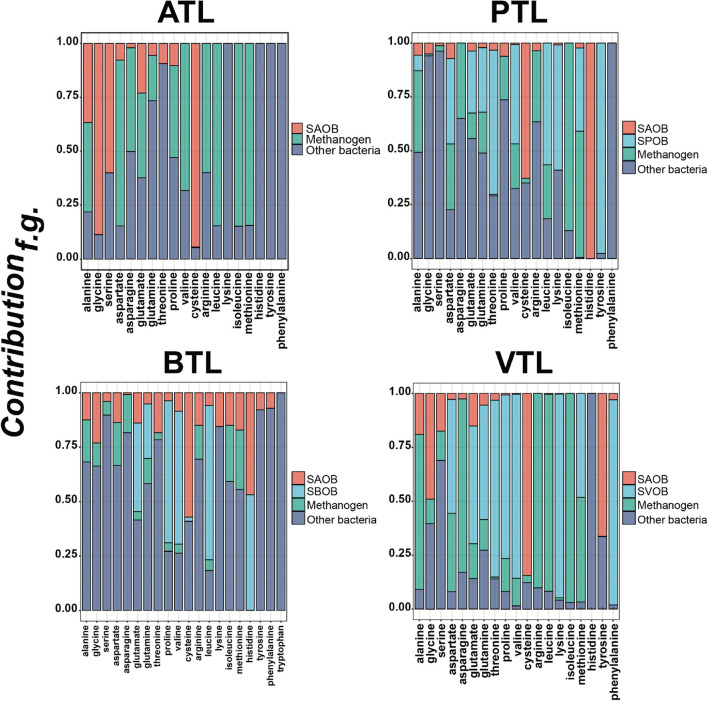
The contributions of different functional groups to amino acid synthesis in each chemostat. The ATL, PTL, BTL, and VTL represent the thermophilic methanogenic chemostats supplemented with acetate, propionate, butyrate, and isovalerate as sole carbon source, respectively. We quantified the relative contribution of AA synthesis of each functional group by defining *Contribution_*f.g.*_.* Details on the definition are described in “Materials and Methods” section. More details about the contributions of different functional groups to the synthesis of each AA are shown in Supplementary Figure 7.

We next focused on the second point of our hypothesis, that is, to test whether the strategies of AA synthesis of the members in each function group were correlated with its energy availability. In general, the relative contributions of different functional groups to each AA were largely varied (Figure 5 and Supplementary Figure 7). SAOBs and methanogens made major contributions to the synthesis of most AAs in ATL, but their contributions reduced in other chemostats, especially in PTL and BTL. As acetate is the main energy source for SAOBs and methanogens (Zheng et al., 2019; Chen et al., 2020a,b), this phenomenon might be related to the differences in the energy (acetate) availability in different chemostats. Under the same TOC fed to each chemostat, as acetate was supplied as the substrate in ATL, the energy (acetate) availability of the methanogenic group (SAOBs and methanogens) in ATL was higher than that in other chemostats, which enabled SAOBs and methanogens in ATL to maintain the highest activity of AA synthesis. In PTL, BTL, and VTL, acetate was generated as an intermediate of carbon metabolism. Theoretically, one-, two-, and three-molecule acetate can be produced from one-molecule propionate, butyrate, and isovalerate by SPOB, SBOB, and SVOB, respectively (Figure 1). Therefore, the carbon converting ratios of propionate to acetate is 2/3, that of butyrate to acetate is 4/4, and that for isovalerate is 6/5. As a result, the accessibility of acetate in VTL was higher than that in PTL and BTL, which enabled SAOBs and methanogens in VTL to obtain more energy from methanogenic metabolism to maintain the higher activity of AA synthesis than that in PTL and BTL. Similarly, the SPOB, SBOB, and SVOB made important contributions to synthesize several costly AAs (histidine, tyrosine, and phenylalanine), which may also be due to their higher energy availability deriving from the direct degradation of the supplying carbon sources (i.e., propionate, butyrate, or isovalerate). In summary, the AA synthesis ability of these functional groups was affected by their energy availability, consistent with the prediction of our hypothesis.

Moreover, we also found that the noncore functional group contributed to synthesizing every AA, especially playing a leading role in the synthesis of those costly AAs, such as histidine, tyrosine, phenylalanine, and tryptophane (Figure 5). This result suggests that those taxa that do not contribute to core metabolism of anaerobic methanogenesis are indispensable in PG synthesis to support other community members, which may explain why these groups always possess considerable abundance in AMCs (Rivière et al., 2009; Krakat et al., 2011; Tang et al., 2011).

### Strategy of Amino Acid Synthesis Within Different Functional Groups

Despite common features of AA synthesis strategies of the community members in each functional group, our further analyses also indicate that, within a same functional group, strategies of AA synthesis are also considerably different among members (Supplementary Figure 4). In order to explore whether these intragroup differences were also associated with energy availability, we further compared the AA synthesis ability of MAGs within each functional group.

#### Amino Acid Synthesis in Syntrophic Acetate Oxidation Bacterias

**FIGURE 6:**
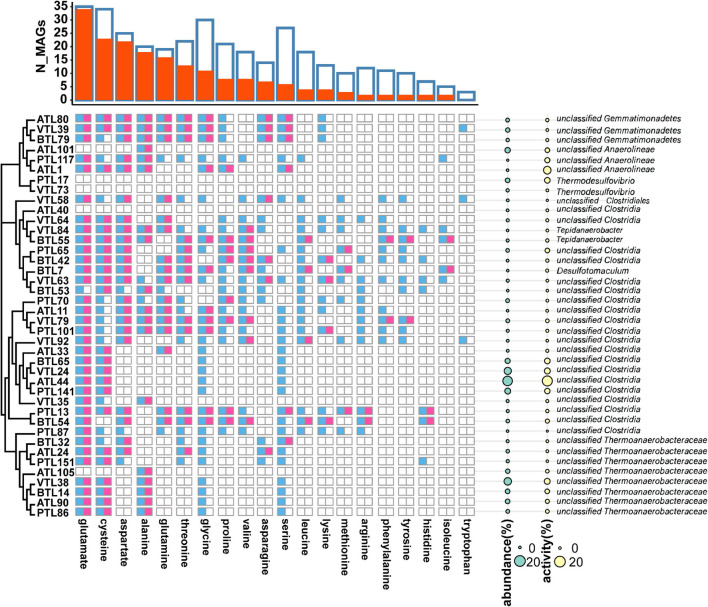
The strategy of amino acid synthesis of syntrophic acetate oxidating bacteria (SAOBs) in different chemostats. The blue squares represent the presence of AA synthesis genes in the corresponding MAGs, whereas the red squares represent the activity of AA synthesis at transcriptomic level. Phylogenetic relationships among these MAGs are shown on the left side. On the right side, the cyan disks represent the abundance of the MAGs, whereas the yellow disks represent their transcriptional activities. The overall landscape of AA synthesis in SAOB MAGs is summarized on the top of the figure. Here, blue frames represent the number of MAGs containing the synthetic genes of the corresponding AA, and the red column represents the number of MAGs that actively transcribed the corresponding AA.

To investigate the factors that affect the strategy of AA synthesis in SAOBs, we first arranged the strategy of AA synthesis of the MAGs of SAOBs according to their phylogenetic relationships (Figure 6). We found that higher-cost AAs (e.g., histidine, isoleucine, tyrosine, and phenylalanine) were synthesized by a small number of MAGs (8 MAGs) that possessed lower relative abundance, whereas the lower-cost AAs could be synthesized by the majority of members with higher abundance. This result indicates that the strategy of AA synthesis within the group of SAOBs was also affected by the biosynthetic cost.

**FIGURE 7:**
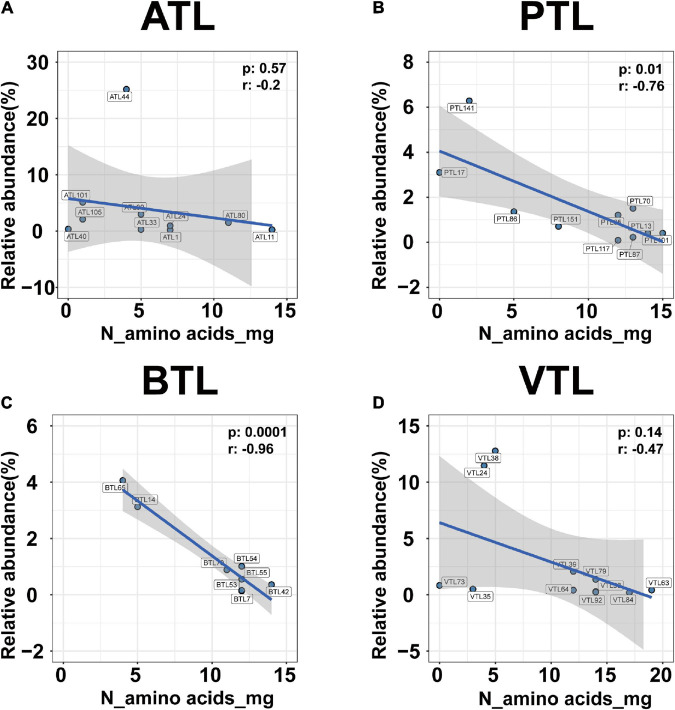
The correlation between the relative abundance and the ability of amino acid synthesis of the MAGs belonging to SAOB at genomic level in ATL **(A)**, PTL **(B)**, BTL **(C)**, and VTL **(D)**. The ATL, PTL, BTL, and VTL represent the thermophilic methanogenic chemostats supplemented with acetate, propionate, butyrate, and isovalerate as sole carbon source, respectively. The “N_amino acids_mg” represents the number of amino acids that can be synthesized by each MAG at genomic level. The shaded area represents the 95% confidence region.

In addition, relative abundance of MAGs that possessed genes to synthesize a larger set of AAs was lower than that of MAGs that possess genes to synthesize only few AAs (Figure 6; left). Pearson correlation test further suggested that from a genomic perspective, the number of AAs that an MAG can synthesize was negatively correlated with its relative abundance in all the four AMCs (Figure 7; for ATL, *p* = 0.57, *r* = −0.2; for PTL, *p* = 0.01, *r* = −0.76; for BTL, *p* = 0.0001, *r* = −0.96; for VTL, *p* = 0.14, *r* = −0.47). Similarly, we also found this negative correlation at the transcriptomic level (Supplementary Figure 8). As we mentioned previously, acetate was supplied as the substrate in ATL, and thus, SAOBs in ATL possess higher energy availability. Higher energy availability causes SAOBs with different AA synthesis ability hold similar fitness, which might result in similar relative abundance of SAOBs in ATL (Figure 7A). Similarly, SAOBs in VTL possessed higher energy availability than those in PTL and BTL, which might cause the observed negative correlation being weaker in VTL than that in PTL and BTL (Figures 7B–D). Taken together, energy (acetate) availability determines whether an organism with a specific synthesis strategy (synthesize more AAs) is favored.

#### Amino Acid Synthesis in Methanogens

**FIGURE 8:**
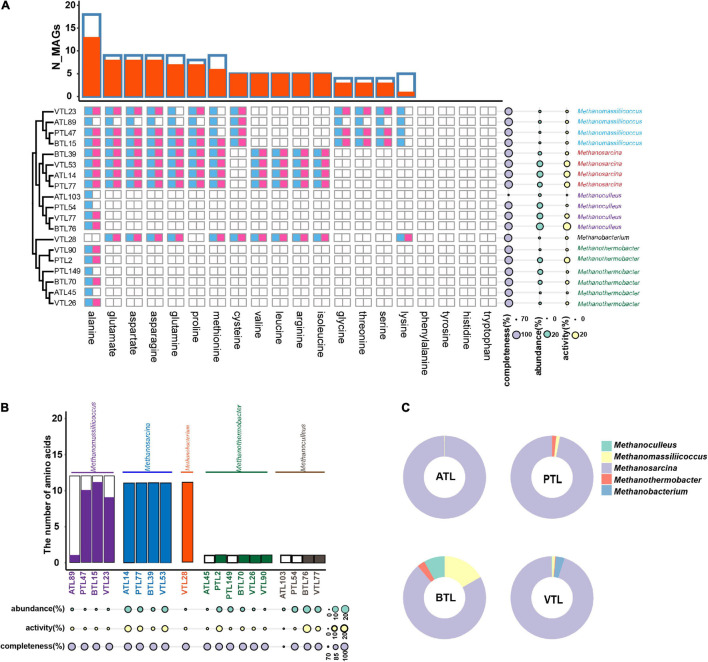
The amino acid synthesis of methanogens in different reactors. **(A)** The blue squares represent the presence of AA synthesis genes in the corresponding MAGs, whereas the red squares represent the activity of AA synthesis at transcriptomic level. Phylogenetic relationships among these MAGs are shown on the left side. On the right side, the cyan disks represent the abundance of the MAGs, whereas the yellow disks represent their transcriptional activities. The overall landscape of AA synthesis in methanogen MAGs are summarized on the top of the figure. Here, blue frames represent the number of MAGs containing the synthetic genes of the corresponding AA, and the red column represents the number of MAGs that actively transcribed corresponding AA. **(B)** Summary of the AA synthesis ability of different methanogen taxa. The black frame represents the number of amino acids that the corresponding MAG can synthesize at genomic level, whereas the color-filled column represents the number of amino acids, of which the synthetic genes were actively transcribed in the corresponding MAG. **(C)** Comparison of relative AA synthesis activity (*RA*_*a.a*_) of methanogens from different genera in each chemostat. The ATL, PTL, BTL, and VTL represent the thermophilic methanogenic chemostats supplemented with acetate, propionate, butyrate, and isovalerate as sole carbon source, respectively.

We then turned to investigate the factors that affect the strategy of AA synthesis in methanogens. As shown in Figure 8, all methanogens in our four AMCs did not contain complete gene set encoding the enzyme of synthesizing those costly AAs (i.e., phenylalanine, tyrosine, histidine, and tryptophan), which indicate that the biosynthetic cost of AAs also had significant impacts on the AA synthesis strategies of methanogens.

Interestingly, MAGs belonging to *Methanosarcina* could synthesize more kinds of AAs and showed higher activity at transcriptomic level than other methanogens (Figures 8B,C). We previously found that, in our AMCs, whereas other methanogen taxa, such as *Methanoculleus* and *Methanothermobacter*, produced methane only by hydrogenotrophic pathway, *Methanosarcina* produced methane through both hydrogenotrophic and acetotrophic pathways (Zeng et al., 2021; Figure 1). In a previous study, we found that *Methanosarcina* mostly obtain energy through the acetic acid pathway (Zeng et al., 2021). As acetate was more available than hydrogen for the methanogens (because acetate was supplied as substrate or as a main intermediate), *Methanosarcina* could gain more energy from its methanogenic metabolism, possessing higher energy availability. Moreover, MAGs belonging to *Methanoculleus* in our thermophilic AMCs exhibited different AA synthesis strategies with those in mesophilic AMCs reported previously (Embree et al., 2015). While *Methanoculleus* in those AMCs could synthesize six AAs, including glutamate, glutamine, asparagine, aspartate, glycine, and serine, *Methanoculleus* in our AMCs could only synthesize alanine (Figure 8A). Through calculation of Gibbs free energy, we found that less energy could be generated in hydrogenotrophic pathway in thermophilic condition than that in mesophilic condition (Supplementary Table 7). Therefore, *Methanoculleus* possessed lower energy availability in thermophilic condition, which might result in less capability of AA synthesis. In summary, these results suggest that strategies of the AA synthesis of methanogens are also largely affected by energy availability.

Furthermore, we observed a strong AA synthesis complementarity between MAGs belonging to *Methanosarcina* and those belonging to *Methanomassiliicoccus* (Figure 8A). *Methanosarcina* and *Methanomassiliicoccus* are frequently observed to co-occur in anaerobic methanogenesis systems (Wang et al., 2019; Wang F. et al., 2020), including our AMCs (Zheng et al., 2019; Chen et al., 2020a,b). Previous studies suggest that this co-occurrence is possibly due to the potential methyl supply of *Methanosarcina* to *Methanomassiliicoccus* (Dong et al., 2017). Here, in our studies, we proposed that the complementarity in PG synthesis and the associated metabolic interdependency might be another reason for their co-occurrence.

#### Amino Acid Synthesis in Noncore Functional Bacteria

As the noncore functional bacteria held a considerably high abundance and made essential contributions to the AA production in our AMCs (Figure 5 and Supplementary Figure 9), we analyzed the factors that affect the strategy of AA synthesis in these MAGs. At phylum level, these MAGs were mainly classified into Proteobacteria, Planctomycetes, Chloroflexi, Bacteroidetes, Firmicutes, and Actinobacteria. The AA synthesis ability of MAGs within this group was largely varied. The MAGs with relatively higher AA synthesis ability were mainly from Firmicutes, Proteobacteria, and Chloroflexi (Supplementary Figure 9). We found that 20 of these MAGs contained genes encoding acetyl-coenzyme A synthetase (Acs), acetate kinase (Ack), and phosphate acyltransferase (Pta), and their encoding genes were also highly transcribed (Supplementary Figure 9 and Supplementary Table 8). These enzymes are reported to catalyze the conversion of acetate to acetyl-CoA. Higher expression of these genes suggested that these MAGs could directly obtain energy from the metabolism of acetyl-CoA, resulting in higher energy availability of them than other noncore functional bacterial MAGs. Correlation analysis further showed that the AA synthesis ability of the “noncore” MAGs is significantly positively correlated with their metabolic activities of converting acetate to acetyl-CoA (Supplementary Table 9). We thus speculated that the strategies of AA synthesis in these noncore functional bacteria were also affected by energy availability.

## Discussion

Here, we systematically probed the AA synthesis strategies of community members in four AMCs. We found that energy availability and energy cost of AA synthesis are key drivers to shape the abilities of AA synthesis in AMCs.

### Extending the Framework of the Black Queen Hypothesis

The BQH provides an explanation for evolution of metabolic interdependency at community level. The basic framework of BQH states that the benefit of not carrying a public function is determined by the saving energy that results from not performing the function. In the recent models and experiments, this benefit was formulated by roughly assessing the energy cost of the public function. For example, the cost of synthesizing an AA was assessed by the ATPs it would consume in the enzymatic reactions (Akashi and Gojobori, 2002). However, our results suggest that more factors should be included to evaluate this benefit in complex communities.

First, estimating cost by the amount of ATP consumption ignored many aspects involved in AA synthesis. For example, synthesis of glutamate consumes 15.3 ATPs, which is much higher than those of serine and glycine. According to the theory developed in previous studies (Estrela et al., 2016; Mas et al., 2016; Pacheco et al., 2019; Wang et al., 2021), the community members that can synthesize glutamate should be less than the ones that can synthesize serine and glycine. However, in our study, more MAGs synthesized glutamate rather than serine and glycine, which is also the case in other similar investigations (Mee et al., 2014). This phenomenon suggests that the cost of glutamate synthesis might be overestimated. In the estimation by Akashi et al., the ATP consumption of the synthesis of AA precursor (generated from the central carbon metabolism, such as Embden–Meyerhof–Parnas pathway, tricarboxylic acid, and the pentose phosphate cycles) was also taken into account. However, at community level, microorganisms could obtain precursor metabolites directly secreted by other community members (Zengler and Zaramela, 2018). Therefore, the energy cost in precursor metabolites should be prudently considered when evaluating the function cost. However, the accurate and quantitative assessment of the AA synthesis cost still faces obstacles. For example, it is difficult to quantify the exchange of precursor metabolites for AA synthesis among community members and identify all the AA biosynthesis pathways in a given member without comprehensive physiological investigation of it. More efforts should be made to overcome these obstacles in further studies.

Second, our results suggest that community members could also regulate the energy cost of a public function at transcriptomic level. This regulation occurred in the synthesis of two aromatic AAs, tyrosine and phenylalanine, whose encoding genes were considerably highly abundant but lowly transcribed in our systems (Supplementary Figure 6). Previous studies have revealed that the transcription of the related genes is regulated by a repressor, TrpR (Otwinowski et al., 1988). We searched our meta-omics data and found the identified trpR genes were highly active in the four MAGs (ATL5, ATL46, BTL5, and PTL65) that possessed genes to synthesize both tyrosine and phenylalanine (Supplementary Table 10). This result suggests that these MAGs could repress the transcription of these genes to decrease its energy cost. We proposed that this strategy may be, to some extent, better than directly losing the functional genes, by which microorganisms could actively change their metabolic states of PG production when facing fluctuated environmental conditions. Therefore, it is important to consider transcriptional physiology of community members when evaluating the benefit of not carrying a public function in complex communities.

Finally, our results indicate that if a community member could gain more energy in the habitat or niche it occupied, it possessed higher ability to produce more costly PGs. This phenomenon was also observed in other systems. For instance, in oil reservoir, aerobic microorganisms that possessed higher energy availability retained higher ability for synthesizing AAs and vitamins than anaerobic microorganisms (Liu et al., 2018). In the methanogenic system enriched by Zhu et al. (2020), *Coprothermobacter proteolyticus* DTU 632 lacked efficient pathway for electron disposal and the pathway of energy metabolism (acetate catabolism), so relied on other community members for those expensive AAs to reduce the energy burden. Therefore, estimating the benefit of function deficiency in a complex community should not only consider how much energy (ATP) is required for a member to perform a public function, but should also assess how much of the total energy this member can obtain from its overall metabolism. We thus propose that the ratio of essential function cost (*c*) to the overall energy availability of a member (*ea*) is a better indicator to quantify the benefit of function deficiency.

### Implications From Phylogenetic Conservatism of Amino Acid Synthesis Strategy

A central goal in microbial ecology is to reveal the relationship between community composition and the community functioning (Zhou, 2009; Krause et al., 2014; Martiny et al., 2015). The key to achieving this goal is to understand whether the function traits of a microorganism are correlated with its phylogeny (Philippot et al., 2010; Martiny et al., 2013; Louca et al., 2018). Our results indicate that the strategies of AA synthesis of MAGs of SAOB and methanogens in our AMCs were clustered according to their phylogenetic relatedness (Figures 6, 8), despite that these MAGs came from different chemostats initialized with different seedings (Chen et al., 2020a,b). For example, ATL90, BTL14, PTL86, and VTL38 (bacterial MAG) in ATL, BTL, PTL, and VTL adapted totally the same strategy of AA synthesis; that is, they only autonomously synthesized glutamate, cysteine, and alanine. Similarly, in all the four chemostats, *Methanothermobacter* and *Methanosarcina* MAGs (archaeal MAG) could only synthesize alanine, whereas all *Methanosarcina* could synthesize a variety of AAs, including alanine, glutamate, aspartate, glutamine, proline, methionine, valine, leucine, arginine, and isoleucine. This result suggests that the function of AA synthesis in microorganisms is highly phylogenetically conserved and may be mostly shaped by vertical inheritance driven by long-term nature selection, but not by rapid convergent evolution through random gene loss or horizontal gene transfer (Martiny et al., 2013). On the one hand, this result suggests that the evolution of PG (AA) exchange in AMCs is mostly driven by nature selection, such as the adaptive gene loss characterized by BQH (Morris et al., 2012). This observation is different from the cases in several other environments, for example, in the host-associated environments, which is driven by random genetic drift (Nilsson et al., 2005; Kuo et al., 2009). On the other hand, as previous studies also suggest that the traits of energy metabolism (anaerobic methanogenesis) in AMCs are also phylogenetically conserved (Martiny et al., 2013; Evans et al., 2019), we hypothesized that AA synthesis ability of microorganisms in AMCs is coevolved with their core energy metabolism to achieve optimal survival strategy. For microorganisms that can only obtain limiting energy, they save energy from AA synthesis for their better growth/survival. For microorganisms that can obtain more energy from its core metabolism, they may retain more AA synthesis genes to increase its own autonomy, reducing its risk facing against environmental fluctuations. Nevertheless, this hypothesis requires further examination.

### Metabolic Interdependency Affects the Performance of the Anaerobic Methanogenic Communities

Anaerobic methanogenic communities play important roles in treating our waste and driving global carbon cycles (Zhang et al., 2021). In AMCs, syntrophic fatty acid–oxidizing bacteria and methanogens cooperate to perform the conversion of fatty acids to methane, acting as the core functional groups (Nobu et al., 2015). However, it was confusing why the community members that are not involved in the core metabolism were present in the AMCs and how they contribute to the development and function of the AMCs (Ruiz-Sánchez et al., 2019; Wang P. et al., 2020). Our results suggest that those noncore functional bacteria are crucial for producing the costly AAs (tryptophan, tyrosine, histidine, and phenylalanine) that support the survival of the core functional groups. In addition to the AA exchange, several studies also indicate that methanogens also rely on those noncore members for vitamins (Hubalek et al., 2017), another type of PG widespread in natural microbial communities (Rodríguez-Fernández et al., 2018). Therefore, by contributing PGs, those previously thought “noncore” members actually plays a “core” role on the development of the AMCs, which further influences the metabolic efficiency of the AMCs. This finding suggests a novel insight on managing these AMCs for better output: manipulate those “noncore” members to contribute more public secretions; the methanogenic groups may perform better to treat our waste.

In addition, the mode of metabolic interdependency might be one of the causes for the vulnerability of AMCs (De Vrieze et al., 2012; Zhu et al., 2020). Our results suggest that those essential and costly AAs (tyrosine, histidine, tryptophan, and phenylalanine) were produced by only a few community members with lower abundance and activity. These low abundant members may be easily lost when the communities undergo environmental fluctuations. Without supplying the essential AAs, the fitness of the functional groups, especially the methanogens that possess low ability of AA synthesis, would rapidly decrease, resulting in the collapse of the community (Chen et al., 2008). In other words, the production of those costly AAs is highly limited in AMCs. Therefore, artificially feeding these AAs to the system is a potential strategy to increase the stability and robustness of AMCs.

Nevertheless, several studies indicate that community with such mode of metabolic interdependency can also benefit community stability. For example, computational simulation in one recent study found that the community with interdependent pattern has better resistance to nutrient disturbances than the community composed of only the autonomous population that can produce all PGs (Wang et al., 2021). Because the resources that were originally wastefully allocated to produce redundant PGs were saved to fight against the harsh environmental change, energy supply is even more limited in the AMCs, and thus, the energy saving by function loss may be selectively favored at the community level in anaerobic environment during long-term evolution.

### Limitations of This Study

Despite these encouraging findings, we acknowledge four limitations of our study. First, we reconstructed the AA synthesis pathway of MAGs based on the KEGG database and determined the AA synthesis capacity of MAGs based on the expression of the pathway in MAGs. This approach is widely used in recent studies (Embree et al., 2015; Liu et al., 2018; Chen et al., 2020a; Zhu et al., 2020). However, it can be misleading due to the incomplete collection of metabolic pathways in the public databases and the limitations of our current understanding on biological metabolic pathways. Moreover, following classical knowledge, we assumed that the 20 AAs are essential to the growth of all the microorganisms. However, recent studies suggest that several bacteria and archaea grow without providing some of these “essential” AAs. For example, (Price et al., 2018) found that heterotrophic bacteria from 10 different genera annotated as AA auxotroph in IMG^1^ could survive independently without extra AA supplement. Therefore, more culture-dependent experiments are required to advance our understanding on microbial metabolism of AAs and then guide our further analysis of AA exchange in complex microbial communities.

Second, we estimated the potential metabolic exchange of AAs between every two MAGs based on whether these MAGs carried out and expressed the related genes. However, this analysis is not enough to thoroughly understand their interactions. For example, the efficiency of PG exchange is largely affected by the transport of the PG across the cells (Kanzaki and Anraku, 1971), the diffusion rate of the PG (Julou et al., 2013), and the spatial positioning of different members (Allen et al., 2013). While the effects of these factors can hardly be achieved by bioinformatics investigations, culture-dependent studies could be adapted to quantify these effects. For example, combination of FISH and NanoSIMS technologies could be used to visualize the relative positioning of different community members in the AMCs, as well as measuring the distribution of PG *in situ*. We expect that these studies could further advance our understanding on PG exchange in AMCs.

Third, based on previous studies, we proposed a formula $\left(p\propto \frac{ea}{c}\right)$ to depict how the abilities of AA synthesis of a microorganism were determined. Unfortunately, because of the rapidity and complexity of the biological metabolic process, we did not directly quantify energy availability of different microorganisms in our system and also lacked a precise way to measure function costs of the AA synthesis as described previously. Therefore, we did not test our hypothesis from a quantitative perspective. We expect further studies being set out to build the quantitative framework based on our proposed formula.

Finally, although the noncore functional bacteria play important roles in producing several costly AAs, the energy sources of these bacteria are still unclear. Our results suggest that some of these bacteria may acquire energy from metabolizing acetyl-CoA, but the conversion of acetate to acetyl-CoA consumes ATPs. The energy balance of the metabolism still required further investigation. Moreover, many noncore bacteria that did not contain these acetate acetylation genes were also considerably abundant in the AMCs. We hypothesized that these noncore bacteria might also scavenge by-products or the dead biomass of the core functional taxa. However, further experiments are still required to provide direct evidence for these hypotheses.

## Conclusion

In summary, we revealed that metabolic interdependency based on AA exchange is prevalent in AMCs and is fundamental to connect different functional taxa through complex interaction webs. For the first time, we proposed that the strategy of public functions in a member residing in a community is largely influenced by how much energy it could acquire, which is determined by the niche it occupied and its metabolic strategy in specific environmental conditions. This finding shed light on how energy availability acts as a driving force of microbial evolution in complex microbial communities and also provides novel insights on how to manage AMCs and address grand challenges facing against environmental pollution.

## Data Availability Statement

The datasets presented in this study can be found in online repositories. The names of the repository/repositories and accession number(s) can be found below: http://bigd.big.ac.cn/gsa, CRA004311.

## Author Contributions

JY: formal analysis, visualization, and writing–reviewing and editing. YZ: resources, software, and methodology. MW: conceptualization and writing–review and editing. Y-QT: supervision, project administration, funding acquisition, and writing–reviewing and editing. All authors contributed to the article and approved the submitted version.

## Conflict of Interest

The authors declare that the research was conducted in the absence of any commercial or financial relationships that could be construed as a potential conflict of interest.

## Publisher’s Note

All claims expressed in this article are solely those of the authors and do not necessarily represent those of their affiliated organizations, or those of the publisher, the editors and the reviewers. Any product that may be evaluated in this article, or claim that may be made by its manufacturer, is not guaranteed or endorsed by the publisher.

## Footnotes

1
https://img.jgi.doe.gov/
