# Supplementary material for: Energy Availability Determines Strategy of Microbial Amino Acid Synthesis in Volatile Fatty Acid–Fed Anaerobic Methanogenic Chemostats
Source: Front Microbiol. 2021 Oct 4;12:744834. doi: 10.3389/fmicb.2021.744834 (PMC8521154; doi:10.3389/fmicb.2021.744834)
Supplement: Supplementary file 1 [file Data_Sheet_1.docx]

***Supporting Information for***

**Energy availability determines strategy of microbial amino-acid synthesis in volatile fatty acids-fed anaerobic methanogenic chemostats**

Jian Yao, Yan Zeng, Miaoxiao Wang^*^, Yue-Qin Tang^*^

College of Architecture and Environment, Sichuan University, No. 24, South Section 1, First Ring Road, Chengdu, Sichuan 610065, China

^*^Correspondence: Miaoxiao Wang and Yue-Qin Tang

Tel. (fax): +86 28 85990936

E-mail: wangmx2014@pku.edu.cn and [tangyq@scu.edu.cn](mailto:tangyq@scu.edu.cn)

# Contents

**Supplementary methods**

- Metagenomic and metatranscriptomic sample preparation, sequencing, assembly, and binning

**-** Analyses of potential complementarity in AA synthesis between pairs of MAGs.

**-**The code of visualization of the potential amino acid exchange of MAGs in R.

**Supplementary figures**

-**Fig. S1.** A schematic diagram showing the settings of the completely stirred tank reactors used in this study.

-**Fig. S2.** The basic information of the MAGs in the four chemostats used in this study.

-**Fig. S3.** The biosynthesis pathway of each amino acid considered in this study.

-**Fig. S4.** The landscape of amino acid synthesis of all MAGs in the four chemostats.

-**Fig. S5.** A representative diagram showing how the potential amino acids (AAs) exchange between MAGs were depicted in Fig 2.

**-Fig. S6.** The relationship between community-level biosynthesis ability of each amino acid (AA) and its biosynthetic cost in terms of number of phosphates required.

**-Fig. S7.** The contributions of different functional groups to each AA synthesis in each chemostat.

**-Fig. S8.** The correlation between the abundance and the ability of amino acid synthesis of the MAGs belonging to SAOB at transcriptomic level.

-**Fig. S9.** The expression of genes encoding Acs, Ack and Pta in non-core functional bacterial MAGs.

**Supplementary tables**

**-Table S1.** Biochemical reactions performed by different functional groups

**-Table S2.** Locus tags for genes and gene expression level (RPKM and RPKM-NM) involved in amino acids synthesis found in all MAGs from ATL.

**-Table S3.** Locus tags for genes and gene expression level (RPKM and RPKM-NM) involved in amino acids synthesis found in all MAGs from PTL.

**-Table S4.** Locus tags for genes and gene expression level (RPKM and RPKM-NM) involved in amino acids synthesis found in all MAGs from BTL.

**-Table S5.** Locus tags for genes and gene expression level (RPKM and RPKM-NM) involved in amino acids synthesis found in all MAGs from VTL.

**-Table S6.** The contribution index (*ci*) of each MAG.

**-Table S7.** Free-energy changes in methanogenic systems.

**-Table S8.** Locus tags for gene expression level (RPKM and RPKM-NM) of Acs, Ack and Pta found in non-core functional bacteria from thermophilic chemostats.

**-Table S9.** The pearson's correlation test between amino acid synthetic ability and the activity of Acs, Ack and Pta

**-Table S10.** The expression of TrpR operon repressor in MAGs

**-Table S11.** The amino acid synthesis strategy matrix of MAGs in ATL (matrix_1_).

**-Table S12.** The matrix_2_.

**-Table S13.** The potential amino acid exchange relationship between MAGs (matrix_3_)

# Supplementary Methods

**- Metagenomic and metatranscriptomic sample preparation, sequencing, assembly, and binning**

20 ml culture broth was collected and centrifuged at 8000×g for 10 min at 4℃. Total DNA and RNA were extracted from the samples through cetyl trimethyl ammonium bromide (CTAB) method (Griffiths et al., 2000). Metagenomic DNA was sequenced through Illumina HiSeq 2000 platform. The paired-end reads (2 × 150 bp) were trimmed via Trimmomatic v0.36 (Anthony et al., 2014) with a quality cutoff of 30, silding window of 6 bp and minimum length cutoff of 100 bp. The clean Illumina paired-end reads were co-assembled via SPAdes v.3.5.0 (Bankevich et al., 2012), binned by MetaBAT (Kang et al., 2015) and checked for completeness and contamination using CheckM (Parks et al., 2015).

For metatranscriptomics analysis, total RNA was purified via removal of residual DNA by an RNase-free DNase set (Qiagen, Hilden, Germany). Ribosomal RNA was removed from the DNase-treated RNA using Ribo-Zero rRNA Removal Kit (Illumina). RNAseq libraries were constructed using the TruSeq RNA sample prep kit (Illumina) with the standard protocol. The libraries were sequenced on an Illumina HiSeq 2000 platform. The paired-end (2 × 150 bp) metatranscriptomic reads were trimmed as DNA-trimming step described above and mapped to MAGs using the BBMap with the parameters as: minid = 1 (v35.85; 587 http://sourceforge.net/projects/bbmap/).

**-Analyses of potential complementarity in AA synthesis between pairs of MAGs.**

In order to predict the potential complementary relationship in AA synthesis between every pair of MAGs in each chemostat, a digital vector composed of 0 and 1 was constructed to depict the presence (1) or not (0) of the complete synthetic pathway of an AA in each MAG. The digital vectors of all the MAGs in a chemostat was assembled to obtain a matrix (matrix_1_), as shown in Table S11 (take ATL for example). Then, the digital vectors of every pair MAGs were subtracted to obtain two ‘interaction vectors’, which consists -1, +1 and 0 (summarized by the matrix_2_; Table S12; take ATL for example). For example, in the interaction vector of MAG_A_ to MAG_B_, +1 indicates that MAG_A_ potentially supplied the corresponding AA to MAG_B_, while -1 denotes MAG_A_ would obtain the AA from MAG_B_; 0 represented that AA exchange is absent between the two MAGs. The interaction vector of MAG_B_ to MAG_A_ is the reverse ‘interaction vector’ of MAG_A_ to MAG_B_. By counting how many AA a MAG_A_ could contribute to MAG_B_ (that is, counting the number of ‘1’ in IV of MAG_A_ to MAG_B_), as well as how many AA a MAG_A_ could obtain from MAG_B_ (that is, counting the number of ‘-1’ in IV of MAG_A_ to MAG_B_), the ‘contribution strength’ and ‘obtain strength’ of MAG_A_ to MAG_B_ were obtained (see Fig S5, which takes ATL 70 as an example). These analyses were performed between every pair of MAGs in each chemostat (Table S13; take ATL for example). Then, the “circlize” package (Gu et al., 2014) in R (version 4.0.2) was used to visualize the strengths, reflecting the potential amino acid complementary relationship (the R code) between every pair of MAGs. Finally, the ‘contribution strength’ for a given MAG were summarized, divided by the summary of ‘obtain strength’, resulted in the contributing index (*ci*) of this MAGs, reflecting its role as the “contributor” or ‘beneficiaries’ in the community (see Results section).

**-The code of visualization of the potential amino acid exchange of MAGs in R**

library(openxlsx)

library(circlize)

library(viridis)

library(reshape2)

data <- read.xlsx(xlsxFile = "matrix_1_.xlsx",rowNames = T)

unique_name=rownames(data)

sum=NULL

for (i in 1:(length(unique_name)-1) ) {

for (j in (i+1):length(unique_name)){

sum_ij=data[i,]-data[j,]

sum_ij=as.matrix(sum_ij)

rownames(sum_ij)=paste0(rownames(data[i,]),"_",rownames(data[j,]) )

sum=rbind(sum,sum_ij)

}

}

write.csv(matrix_2_,"matrix_2_.csv")

df <- read.xlsx(xlsxFile = "matrix_3_.xlsx",sheet = 1)

df_melt <- melt(df,id.vars = "bin")

colnames(df_melt) <- c('from','to','value')

df_melt$to <- as.character(df_melt$to)

grid.col <- c(ATL84 = "#66C2A5",ATL79 = "#66C2A5",ATL39 = "#66C2A5",ATL38 = "#66C2A5",ATL24 = "#66C2A5",ATL73 = "#66C2A5",ATL56 = "#0D6AAA",ATL23 = "#FC8D62",ATL28 = "#FC8D62",ATL53 = "#FC8D62",ATL26 = "#FC8D62",ATL77 = "#FC8D62",ATL90 = "#FC8D62",ATL60 = "gray",ATL16 = "gray",ATL67 = "gray",ATL81 = "gray",ATL52 = "gray",ATL63 = "gray",ATL58 = "gray",ATL92 = "gray",ATL35 = "gray",ATL12 = "gray",ATL33 = "gray",ATL80 = "gray",ATL93 = "gray",ATL2 = "gray",ATL14 = "gray",ATL72 = "gray",ATL31 = "gray",ATL59 = "gray",ATL62 = "gray",ATL78= "gray",ATL86 = "gray",ATL87 = "gray",ATL88 = "gray",ATL22 = "gray",ATL9 = "gray",ATL29 = "gray",ATL5 = "gray",ATL13 = "gray",ATL47 = "gray",ATL1 = "gray",ATL43 = "gray",ATL46 = "gray",ATL15 = "gray",ATL45 = "gray",ATL30 = "gray",ATL32 = "gray",ATL57 = "gray",ATL20 = "gray",ATL96 = "gray",ATL21 = "gray",ATL17 = "gray",ATL83 = "gray")

grid.col = grid.col,directional = 1,direction.type = c("diffHeight", "arrows"),link.arr.type = "big.arrow"

chordDiagram(df_melt,grid.col = grid.col,transparency = 0.5,directional = 1,direction.type = c("diffHeight", "arrows"),link.arr.type = "big.arrow",annotationTrack = "grid")

circos.track(track.index = 1, panel.fun = function(x, y) {

circos.text(CELL_META$xcenter, CELL_META$ylim[1], CELL_META$sector.index, facing = "clockwise", niceFacing = TRUE, adj = c(0, 0.5))

}, bg.border = NA)

# Supplementary figures


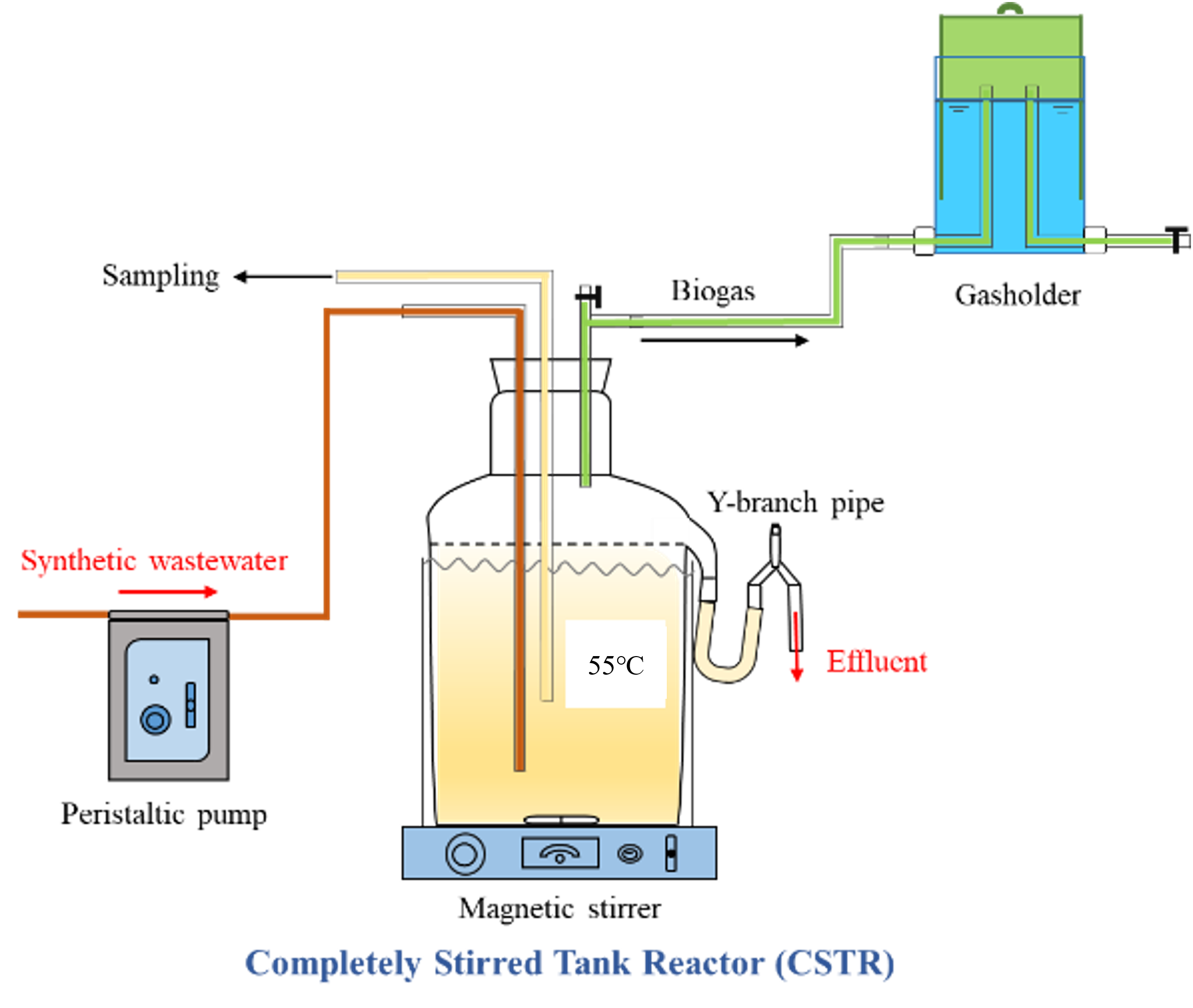


**Fig. S1.** A schematic diagram showing the settings of the completely stirred tank reactors used in this study.


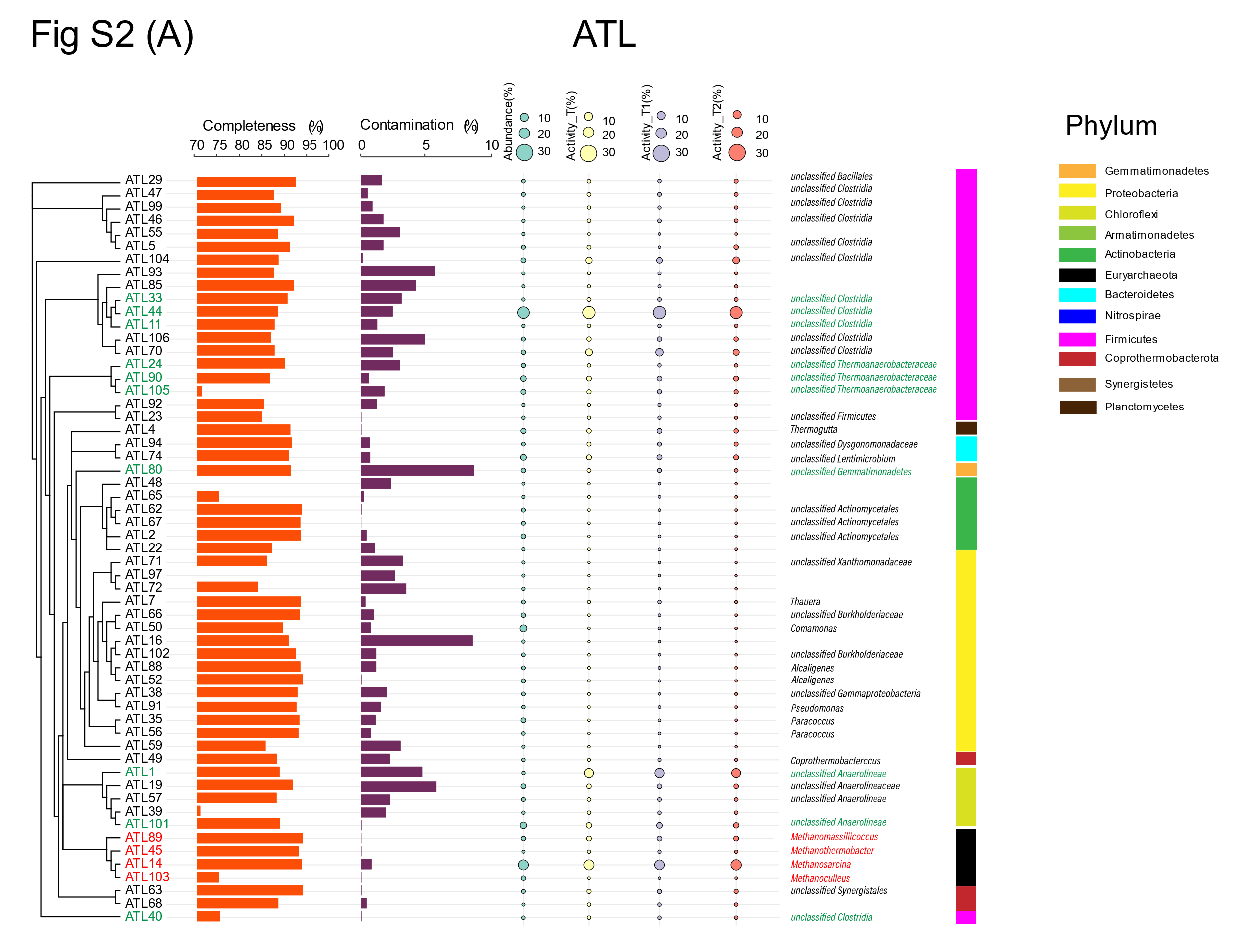


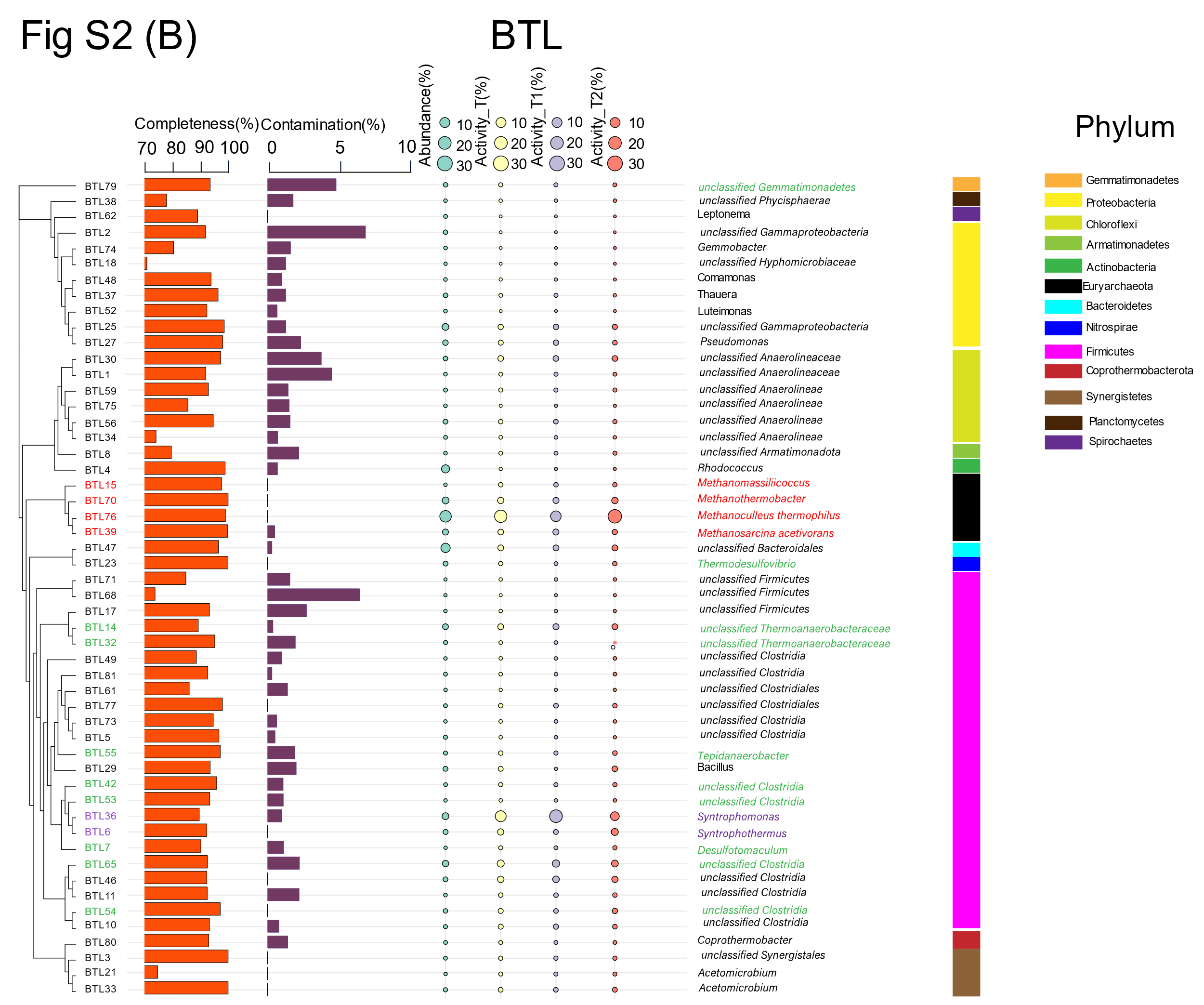


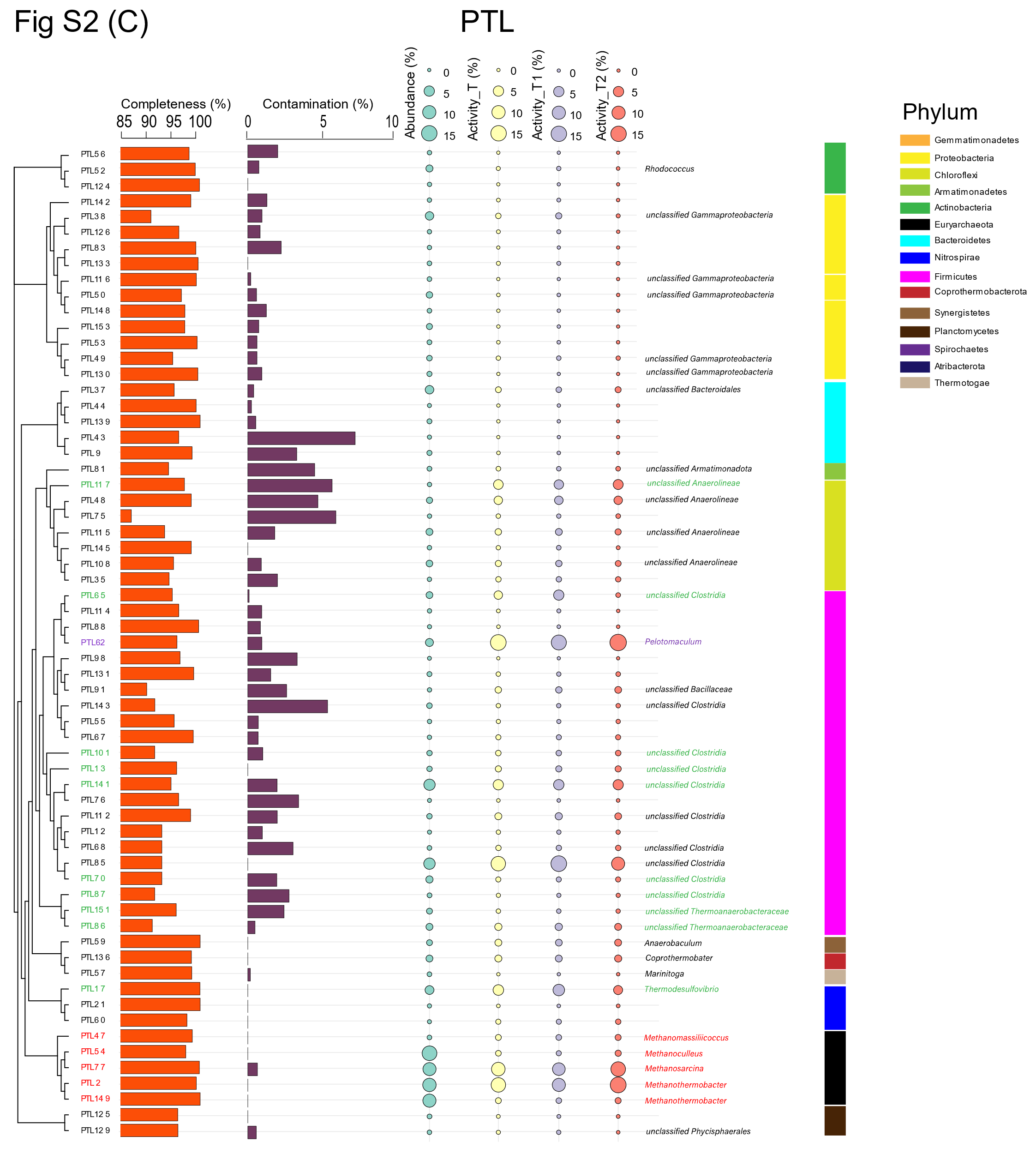


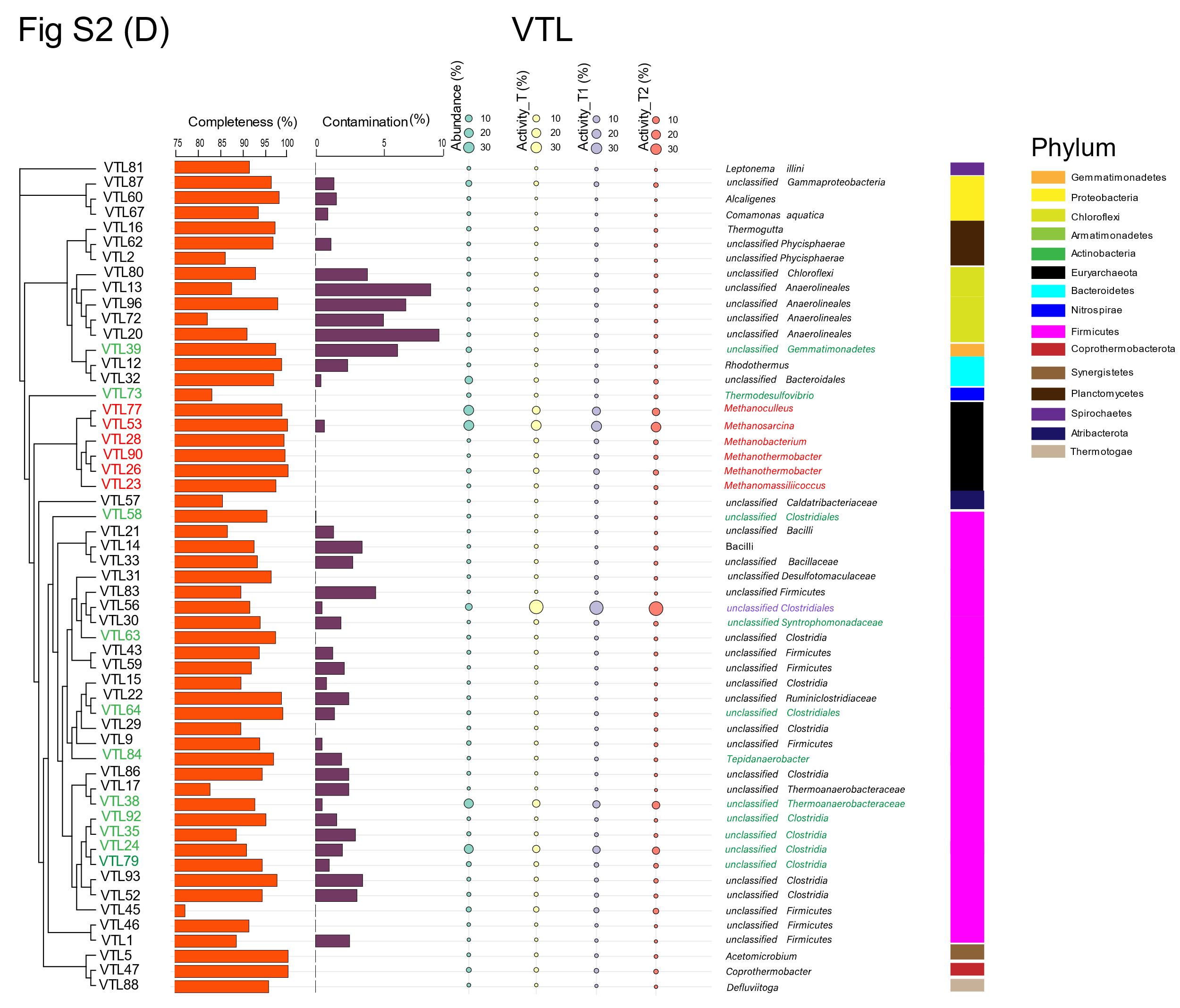


**Fig. S2.** The basic information of the MAGs in the four chemostats used in this study. The ATL, PTL, BTL and VTL represent the thermophilic methanogenic chemostats supplemented with acetate, propionate, butyrate and isovalerate as sole carbon source, respectively. On the top, ‘Abundance’ represents the relative abundance of each MAG estimated from counting the metagenomic reads; ‘Activity_T’, ‘Activity_T1’, and ‘Activity_T2’ represent the estimated activity based on the metatranscriptomic reads (T, total MT reads; T1, MT reads of sampling time 1; T2, MT reads of sampling time 2).


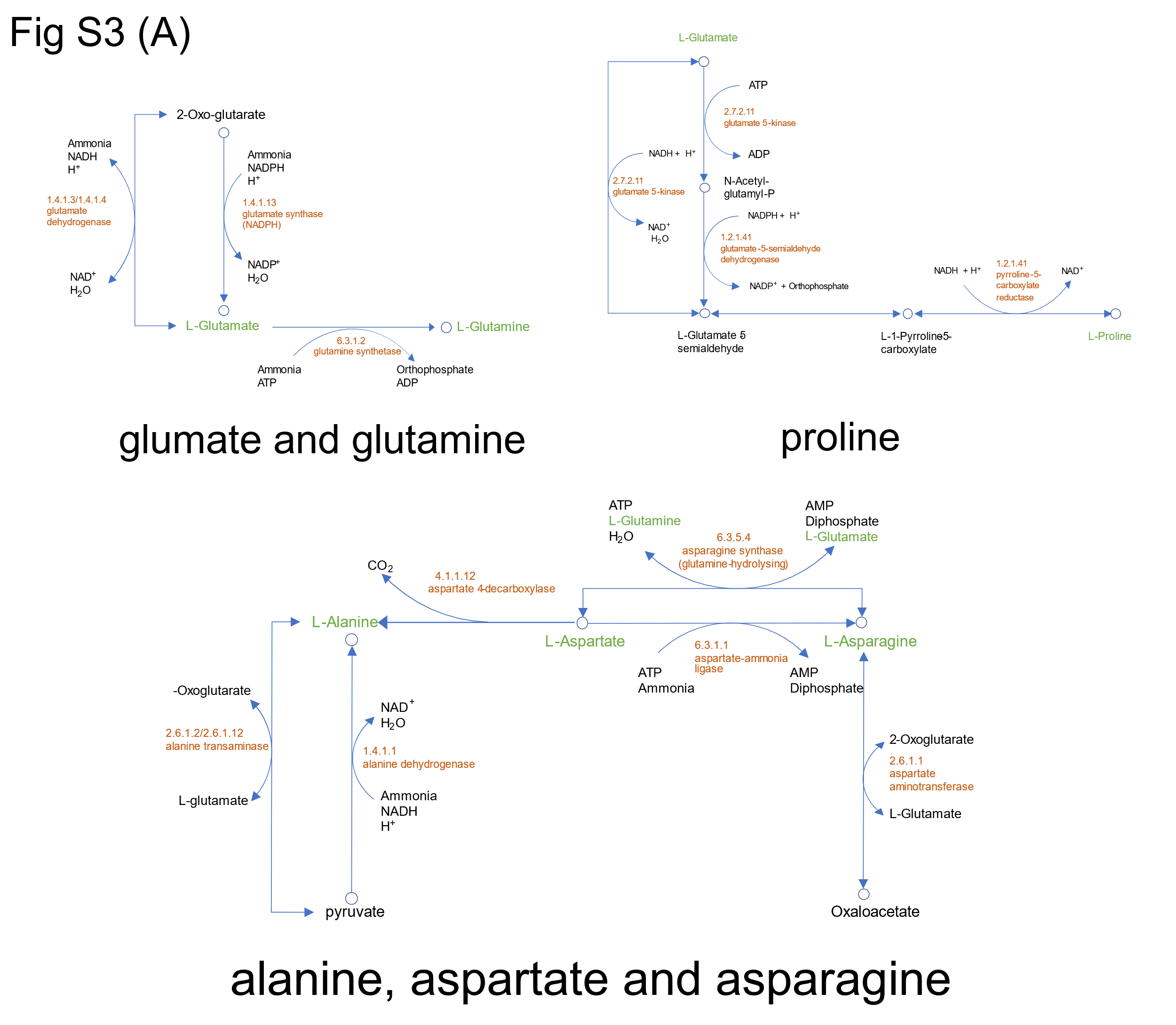


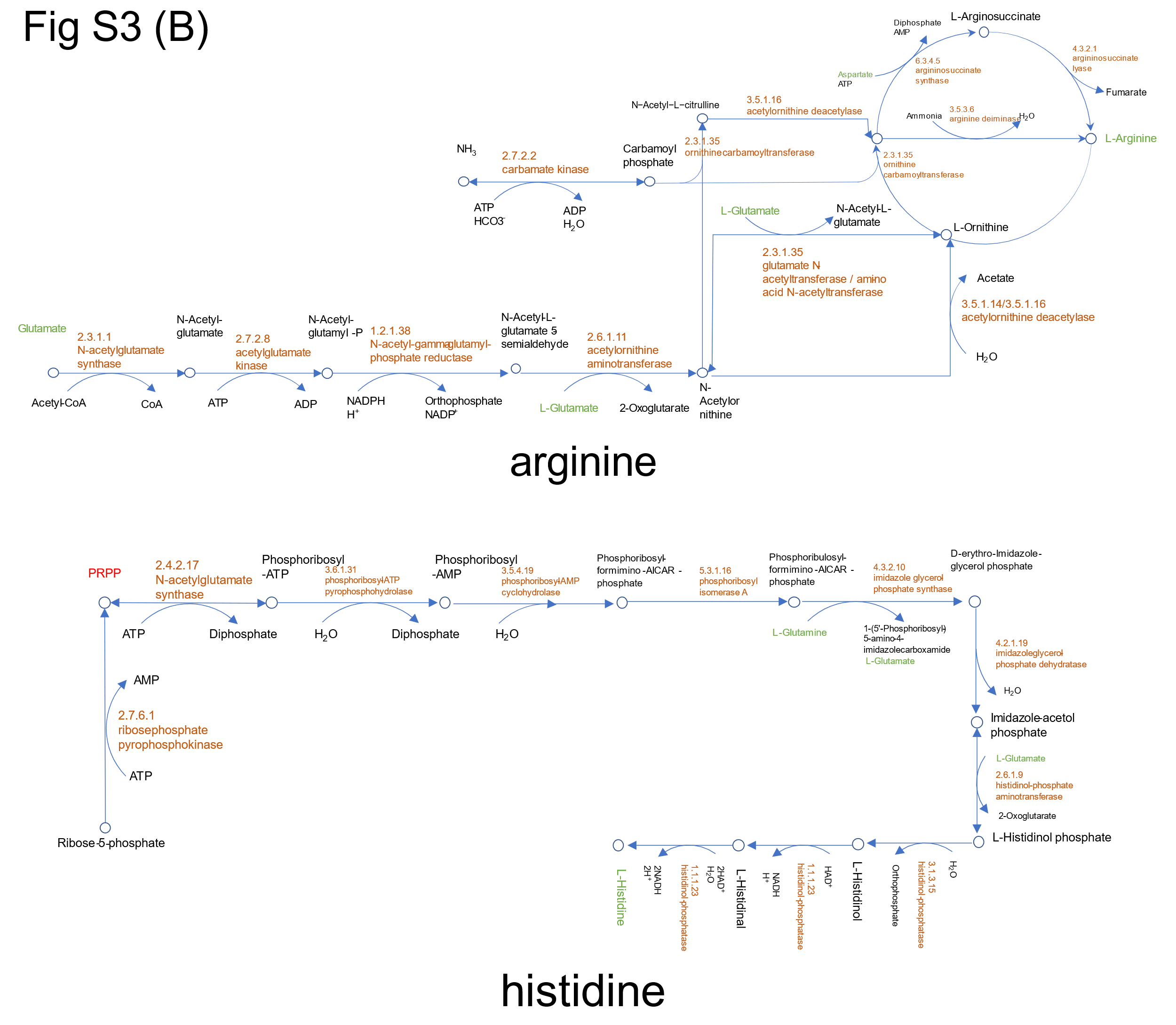


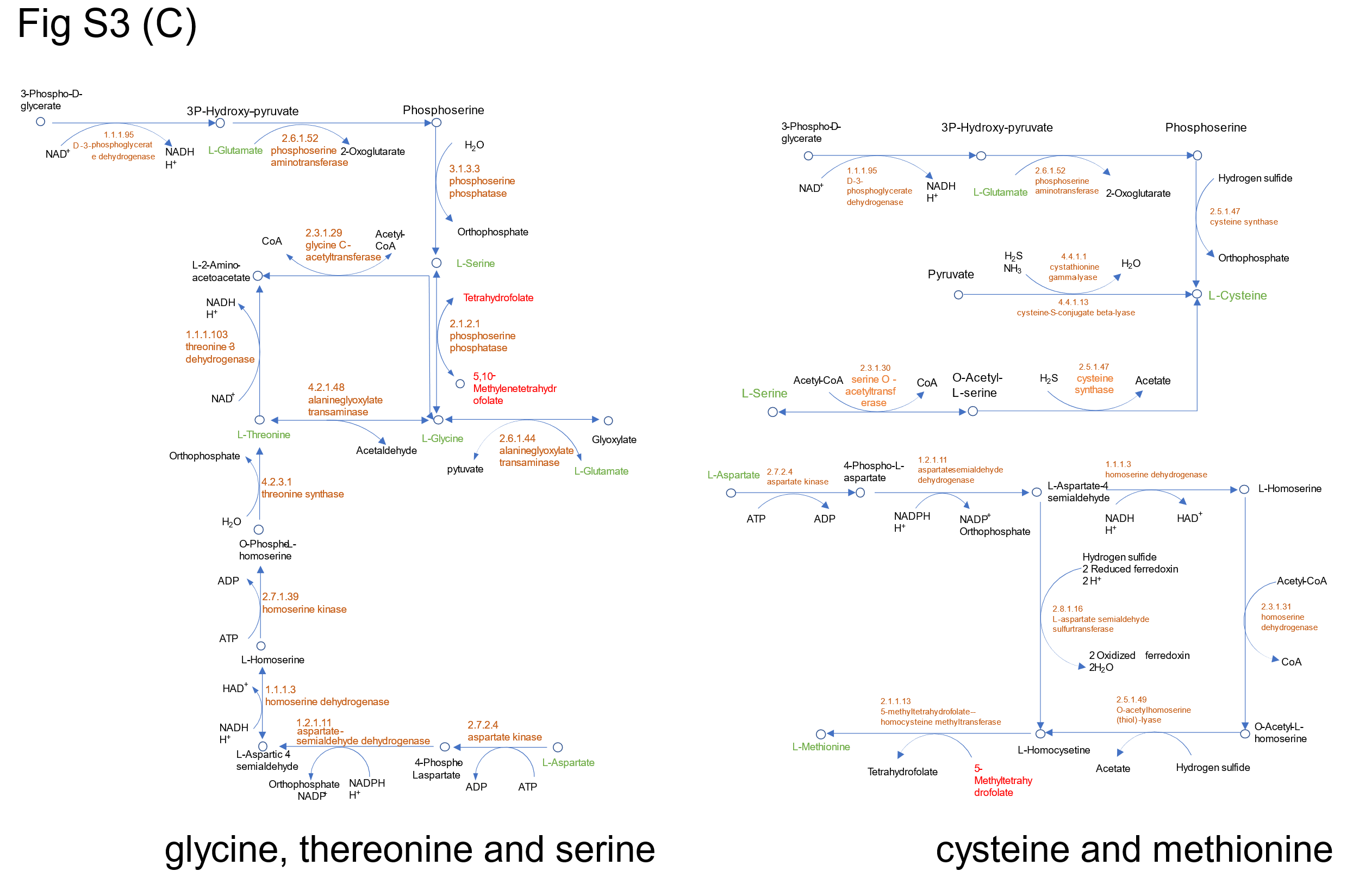


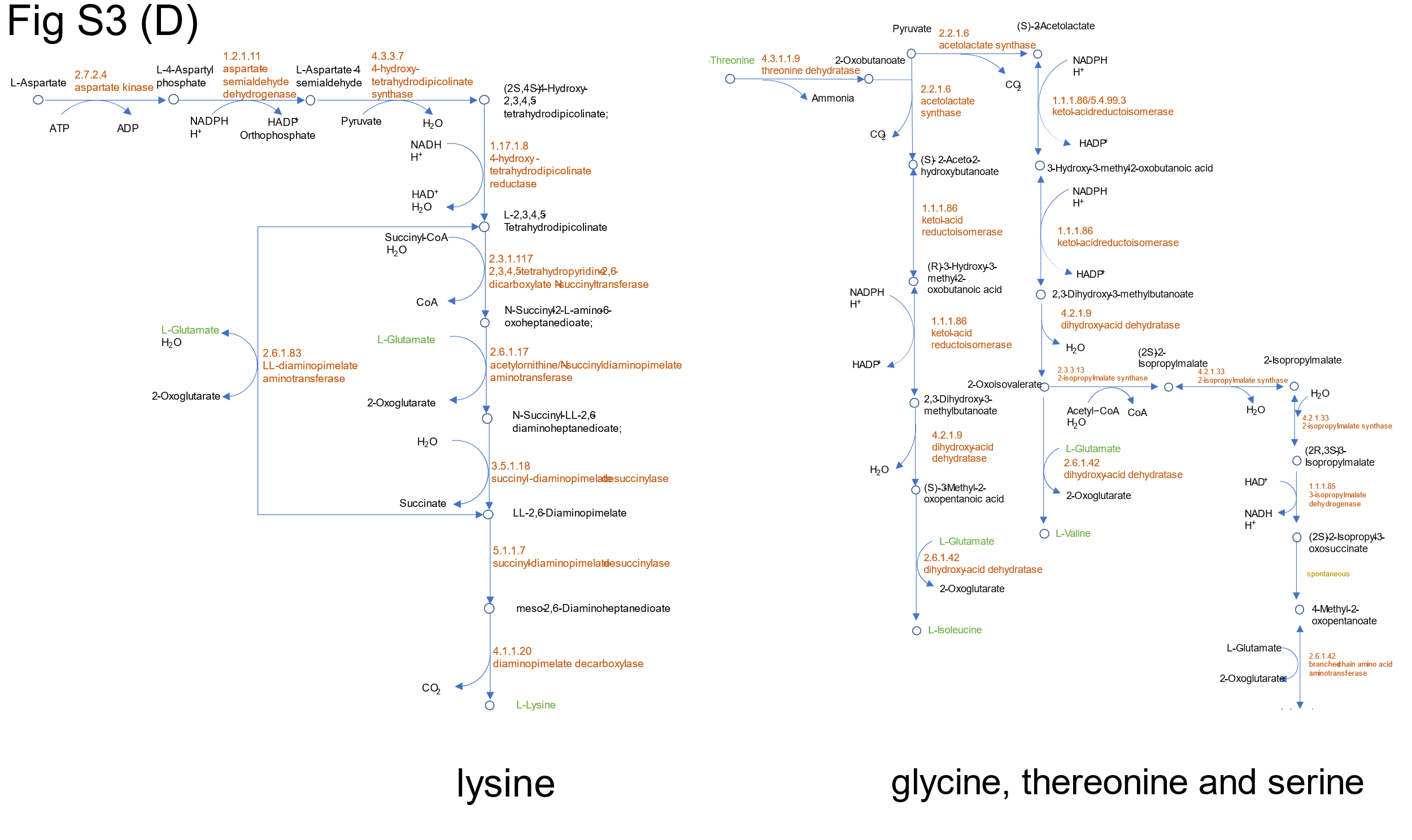


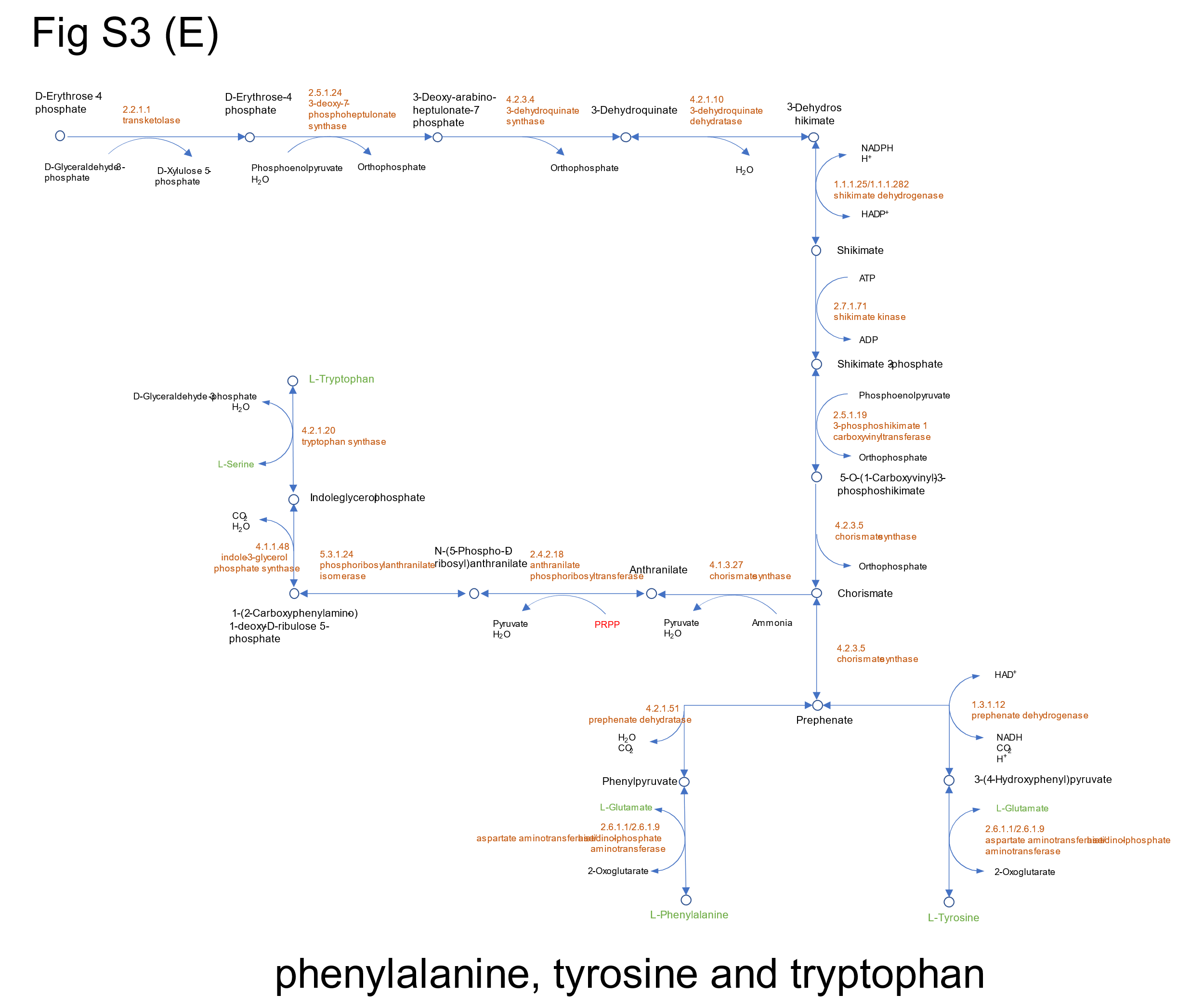


**Fig. S3.** The biosynthesis pathway of each amino acid considered in this study.

The knowledges are based on KEGG pathways (https://www.kegg.jp/kegg/pathway.html#amino).


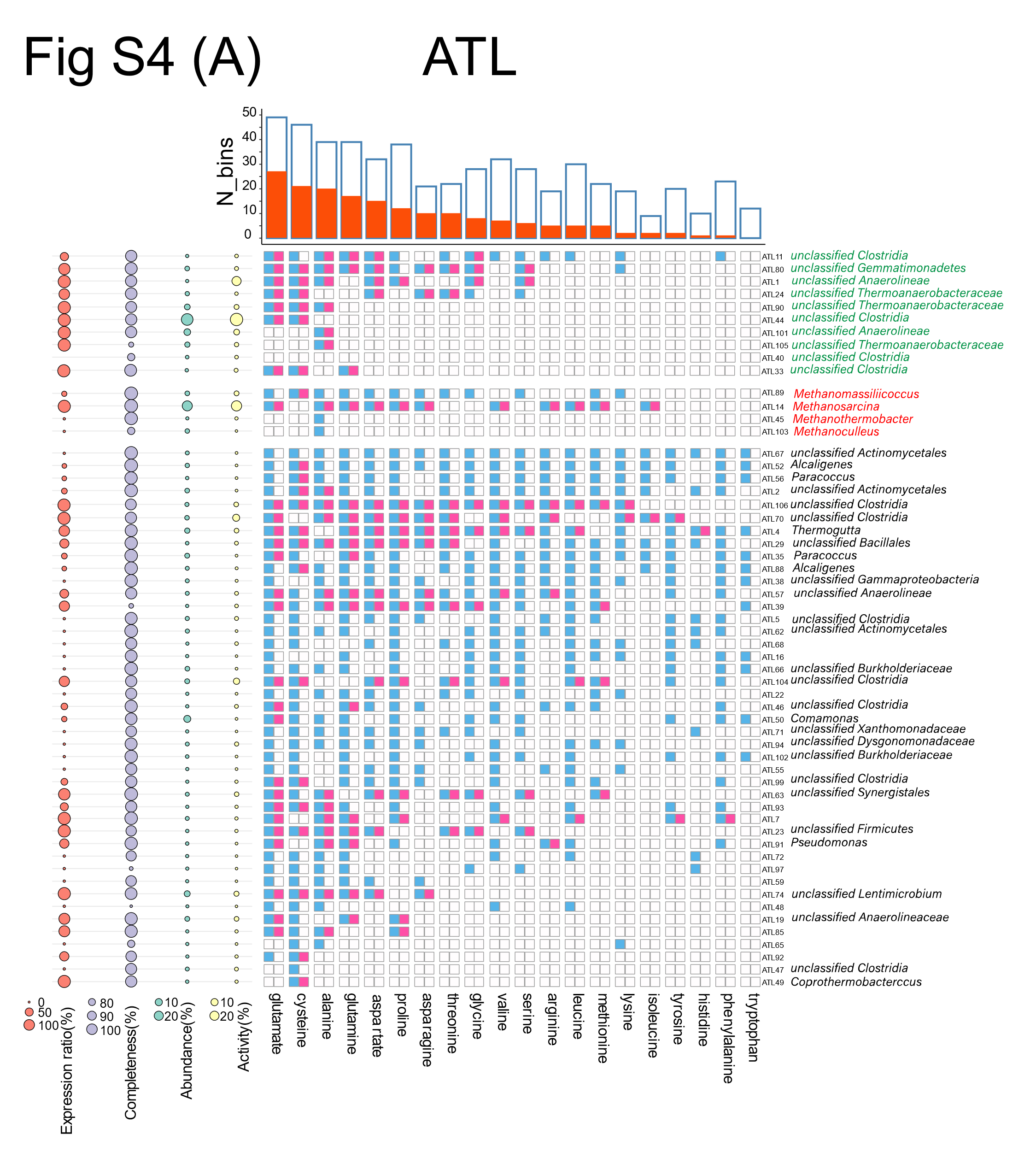


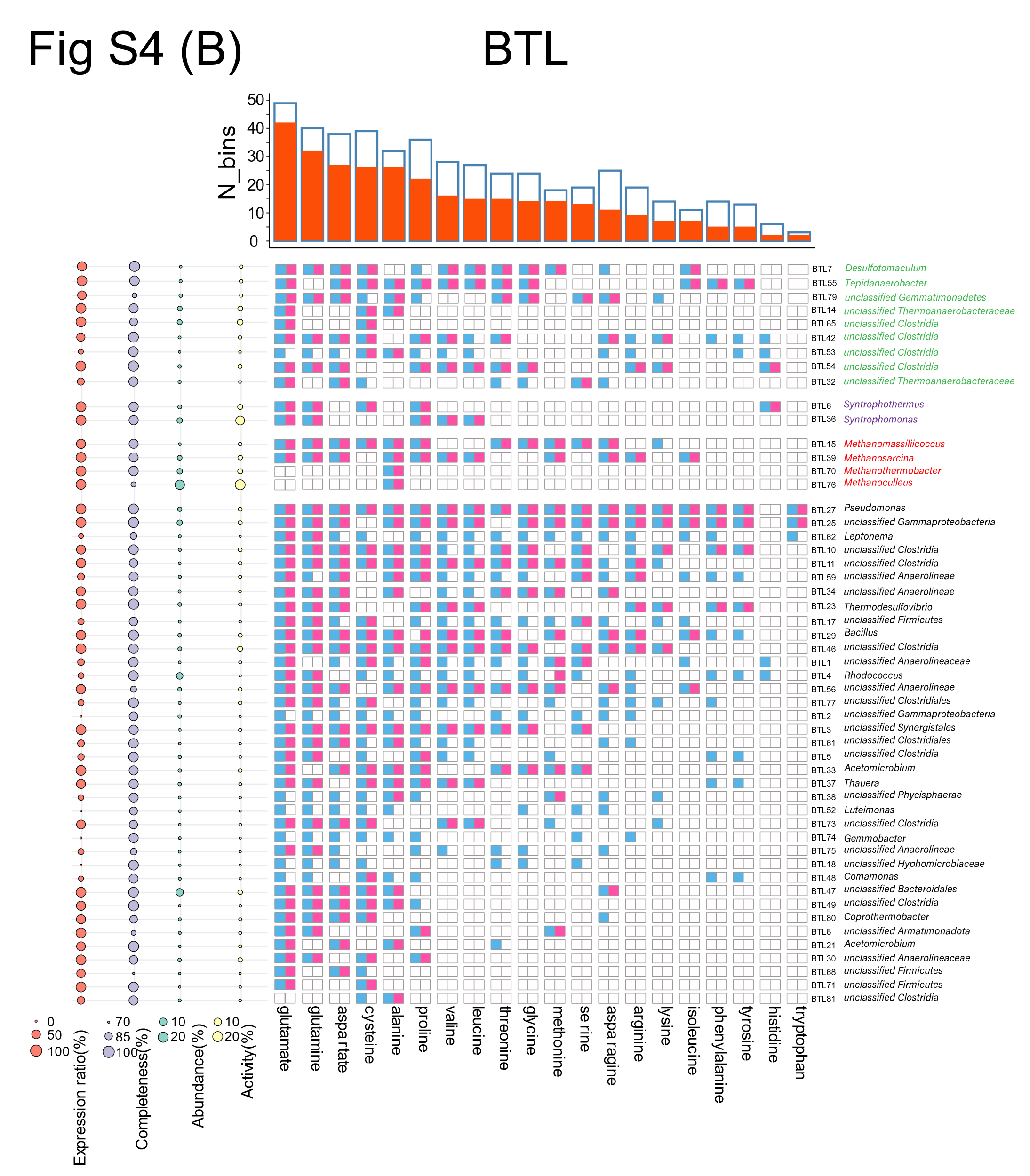


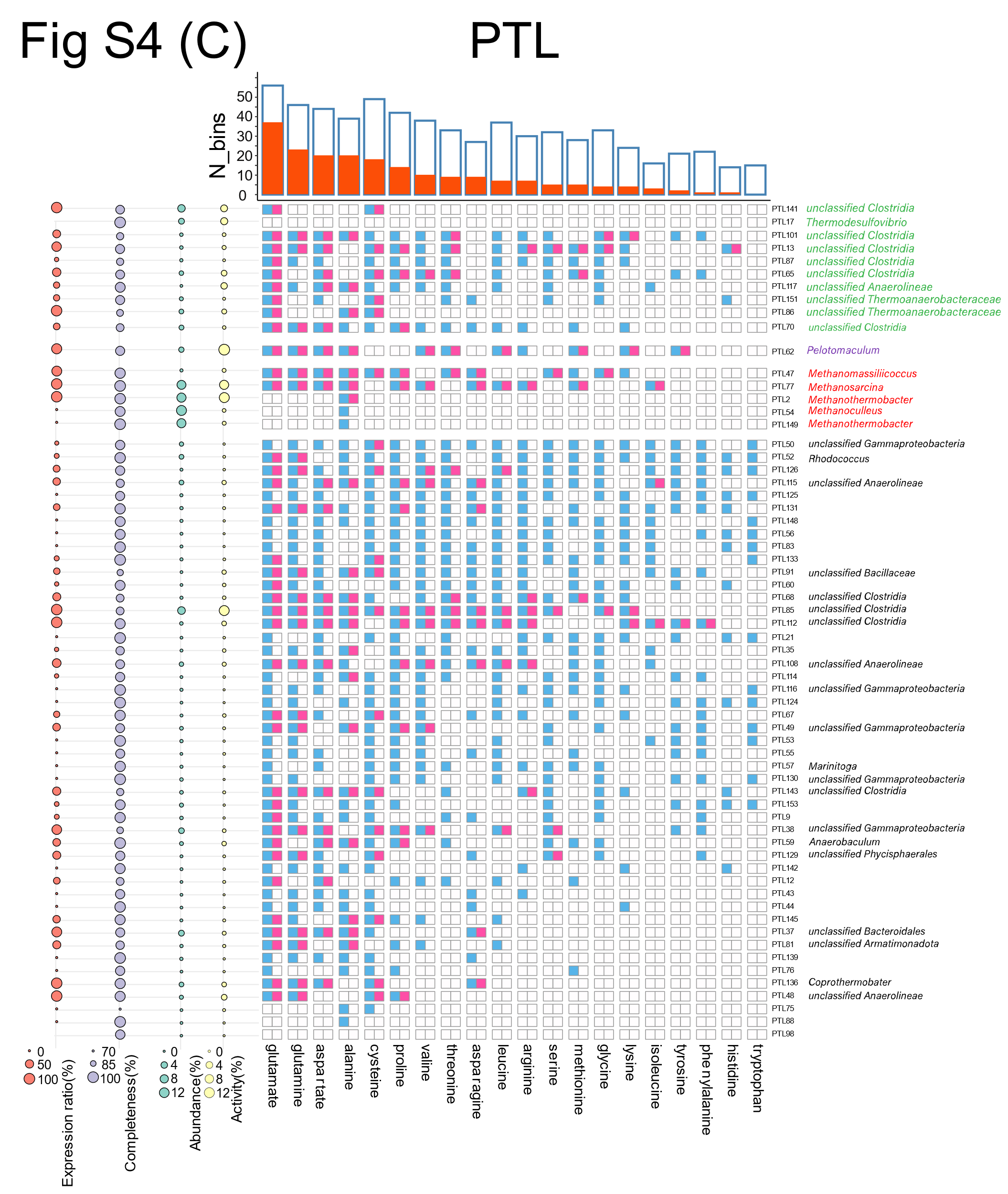


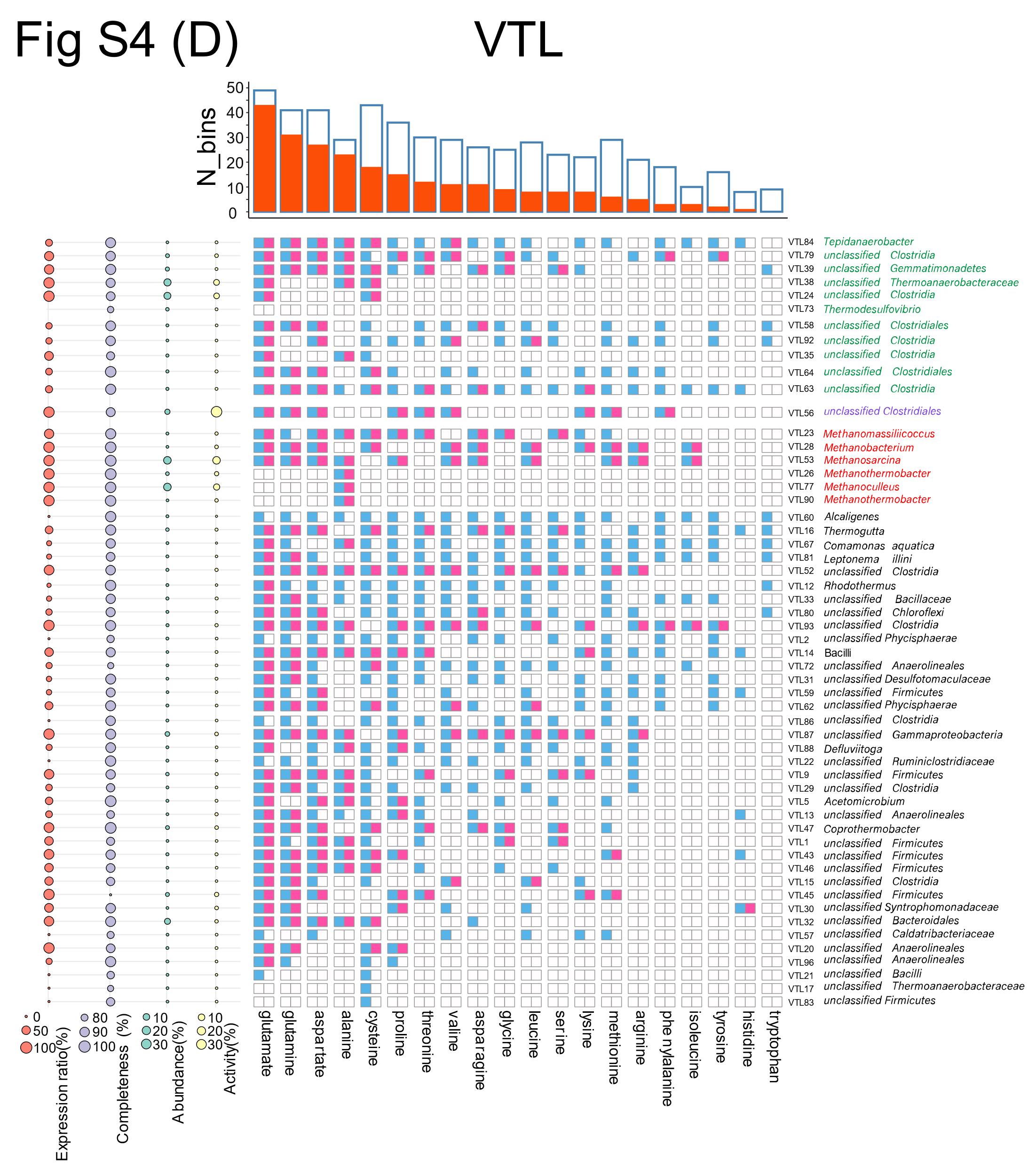


**Fig. S4.** The landscape of amino acid synthesis of all MAGs in the four chemostats. The ATL, PTL, BTL and VTL represent the thermophilic methanogenic chemostats supplemented with acetate, propionate, butyrate and isovalerate as sole carbon source, respectively. The blue squares represent the presence of amino acid (AA) synthesis genes in the corresponding MAGs, whereas the red squares represent the activity of AA synthesis at transcriptomic level. On the right side of figure, methanogens are denoted as red fonts; SAOB are denoted as green fonts; SPOB, SBOB and SAOB are denoted as purple fonts; others bacteria are denoted as black fonts. Expression ratio, relative abundance, activity and completeness of MAGs of each MAG are shown on the left side of the figure. The expression ratio was calculated using the same method as in Fig 4. On the top of the figure, blue frames represent the number of MAGs containing the synthetic genes of the corresponding AA, and the red column represents the number of MAGs that actively transcribed the synthetic genes of the corresponding AA.


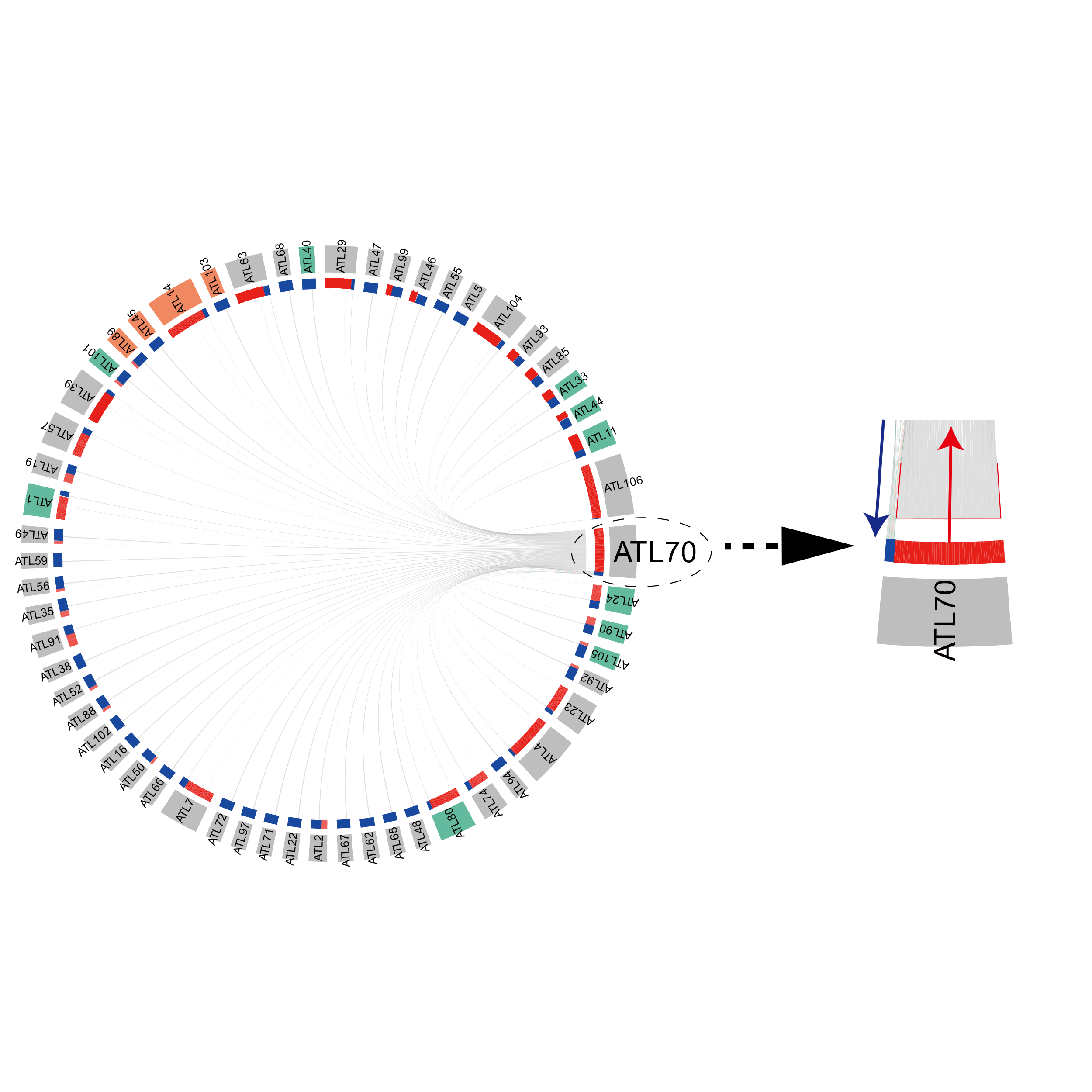


**Fig. S5.** A representative diagram showing how the potential amino acids (AAs) exchange between MAGs were depicted in Fig 2. Setting ATL70 as an example, it could ‘contribute’ a specific number (shown by the link width) of AAs to several other MAGs, denoted by a link started with red patches. Meanwhile it would also ‘obtain’ a specific number (also shown by the link width) of AAs from several other MAGs, denoted by a link end in the blue patches. Consequently, if, for a given MAG, the red patch was wider than the blue one, this MAG tended to contribute more AAs to the community, thus defined as a potential “contributor” (e.g., ATL70). In contrast, if the red patch for a MAG was narrower than the blue patches, this MAG tended to obtain more AAs from other MAGs, thus defined as a potential “beneficiary” (e.g., ATL105 and ATL48).


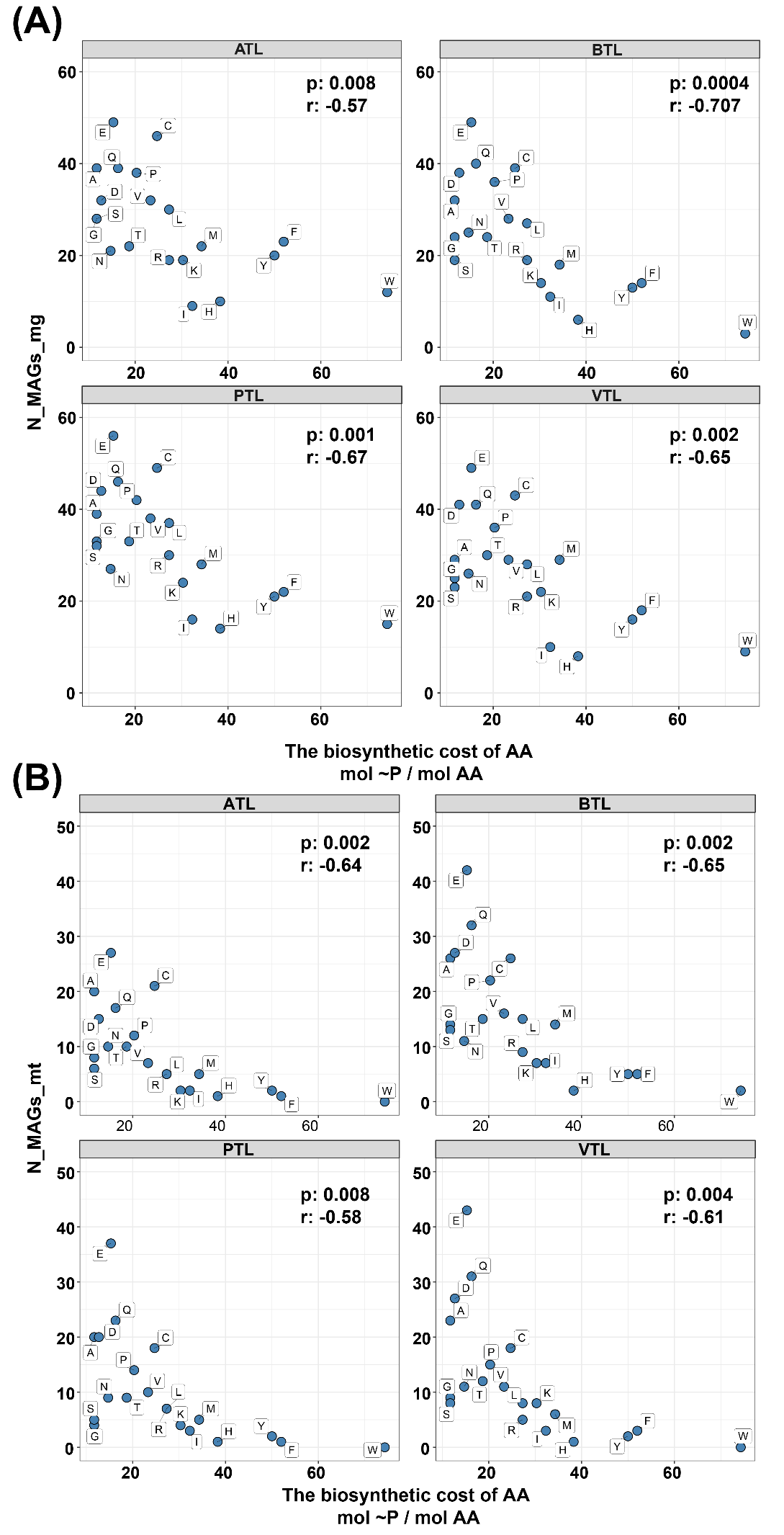


**Fig. S6.** The relationship between community-level biosynthesis ability of each amino acid (AA) and its biosynthetic cost in terms of number of phosphates required. The ATL, PTL, BTL and VTL represent the thermophilic methanogenic chemostats supplemented with acetate, propionate, butyrate and isovalerate as sole carbon source, respectively. N_MAGs_mg represents the number of MAGs that contain all the genes required for synthesizing the corresponding AA. N_MAGs_mt represents the number of MAGs in which the genes responsible for the synthesis of the corresponding AA were actively transcribed. Abbreviations of the AAs: alanine (A), arginine (R), asparagine (N), aspartate (D), cysteine (C), glutamine (Q), glutamate (E), glycine (G), histidine (H), isoleucine (I), leucine (L), lysine (K), methionine (M), phenylalanine (F), proline (P), serine (S), threonine (T), tryptophan (W), tyrosine (Y), valine (V). Correlation analysis were performed by calculated by Pearson correlation method using the cor.test() function of “stats” package (4.0.2) in R (4.0.2).


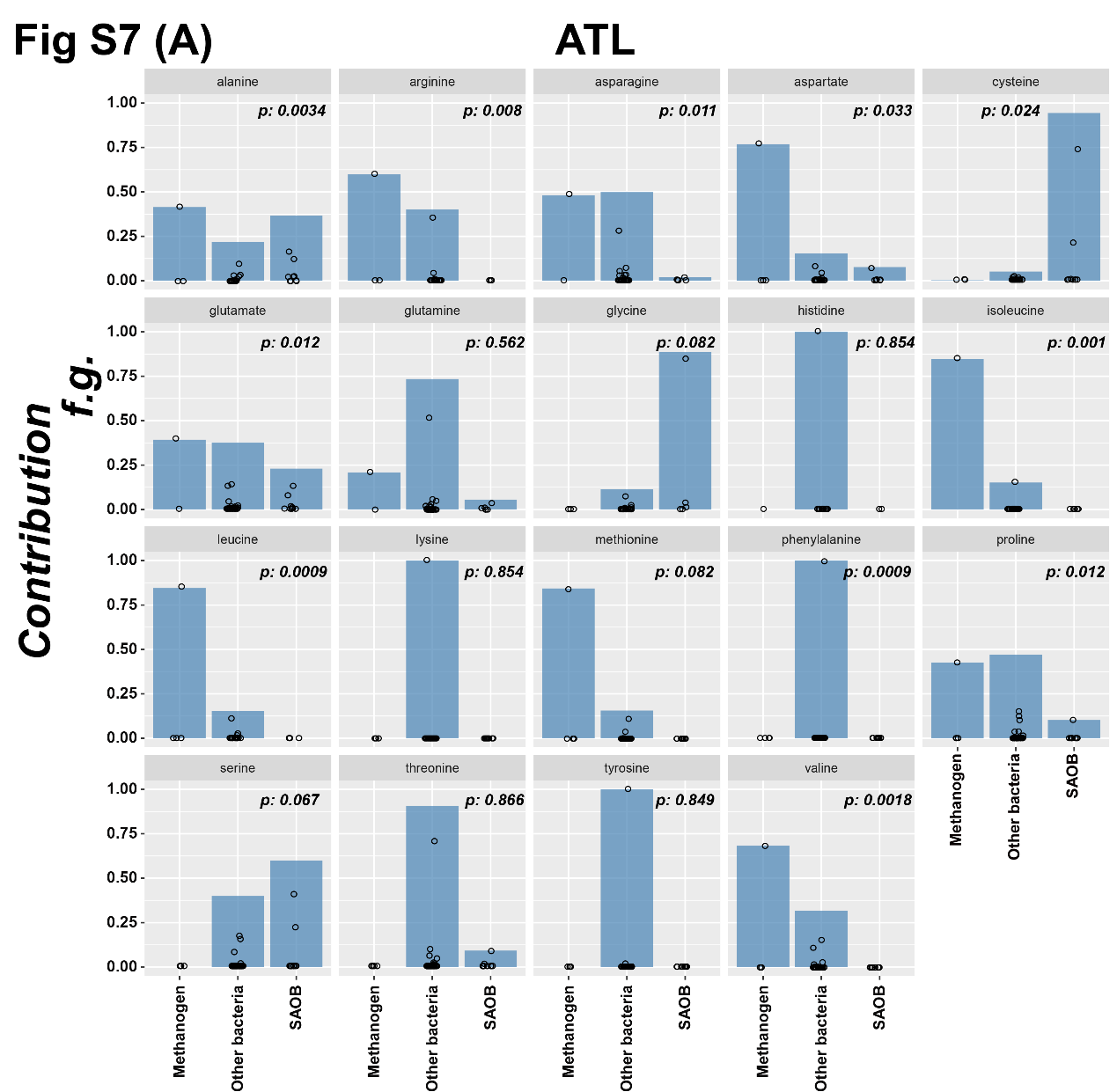


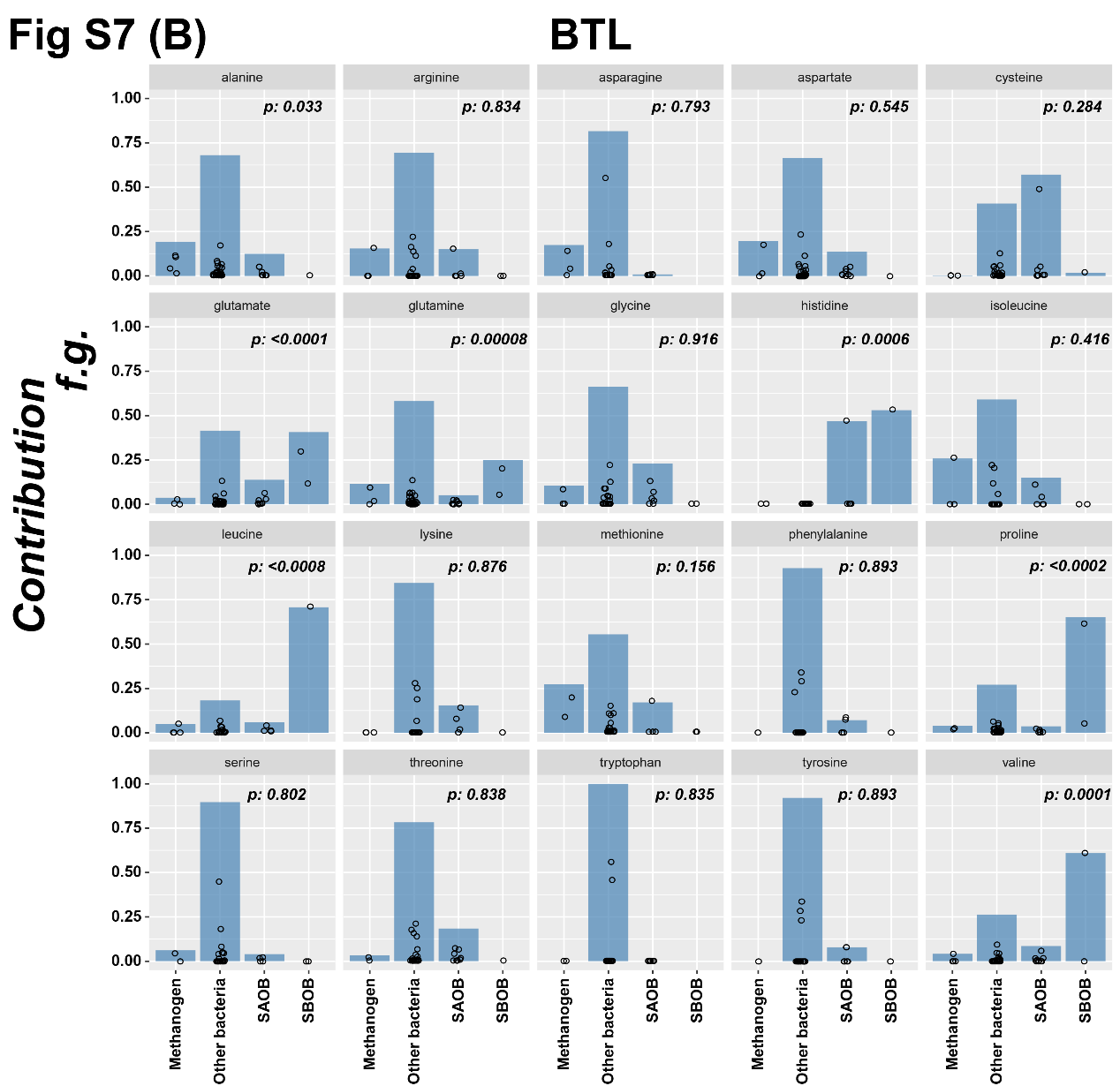


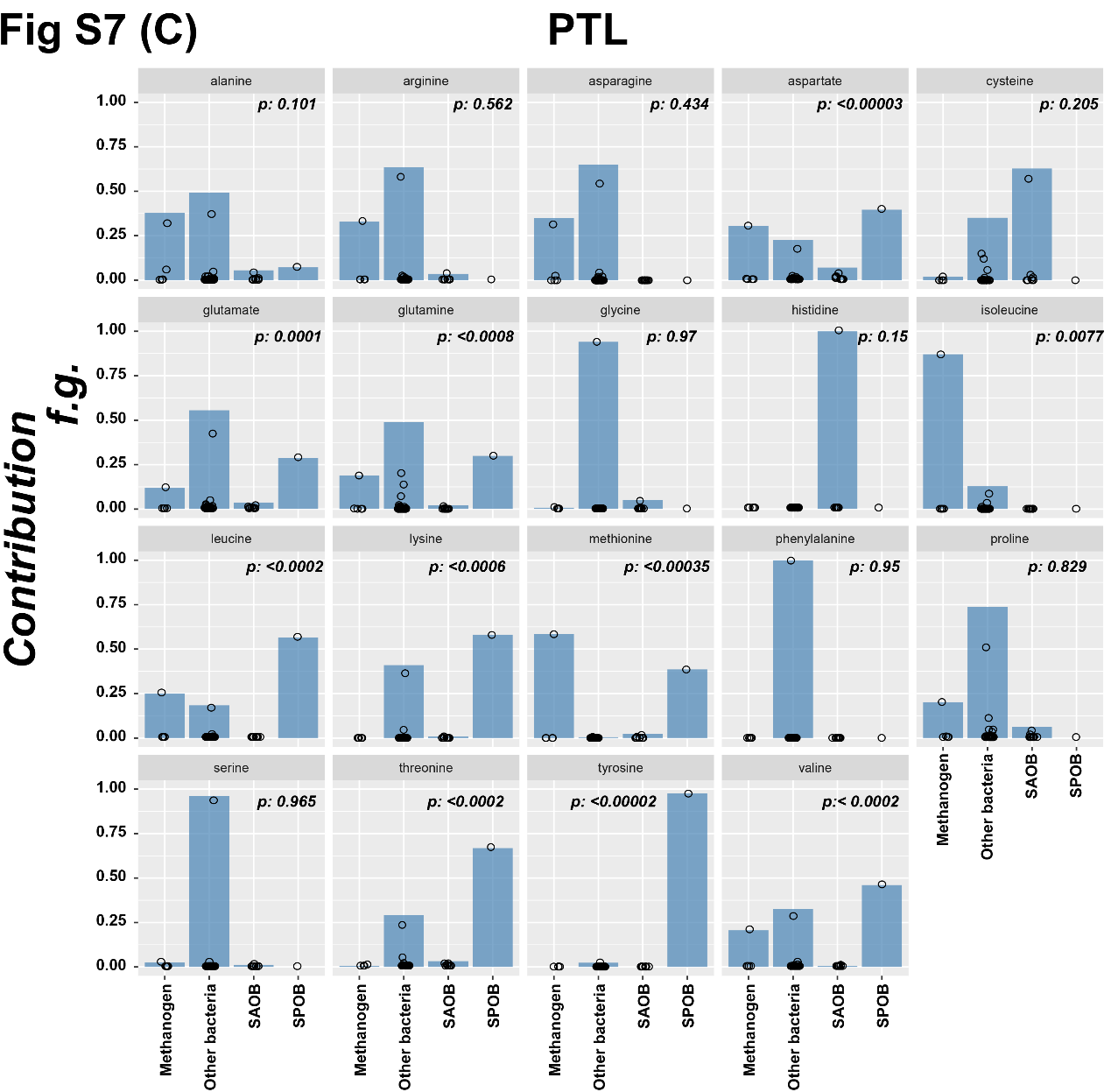


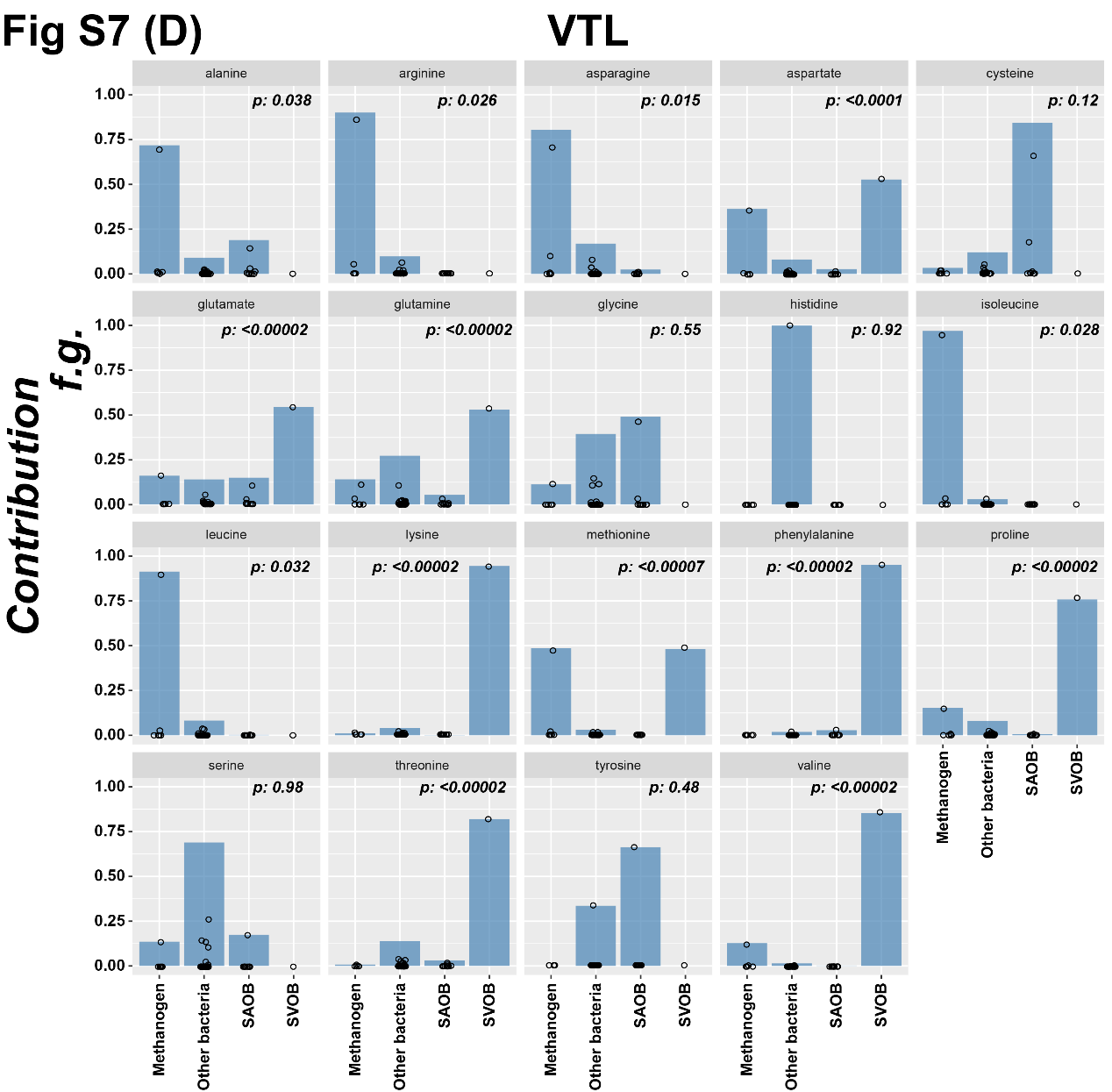


**-Fig. S7.** The contributions of different functional groups to each AA synthesis in each chemostat. In these figures, the ATL, PTL, BTL and VTL represent the thermophilic methanogenic chemostats supplemented with acetate, propionate, butyrate and isovalerate as sole carbon source, respectively. The height of the column represents the sum of the contributions of all MAGs in a functional group to the synthesis of an AA. The hollow points represent the contribution of each MAG to amino acid synthesis. The significance analysis of the difference between different functional groups is performed using the stats package (4.0.2) of R (4.0.2) through ANOVA.


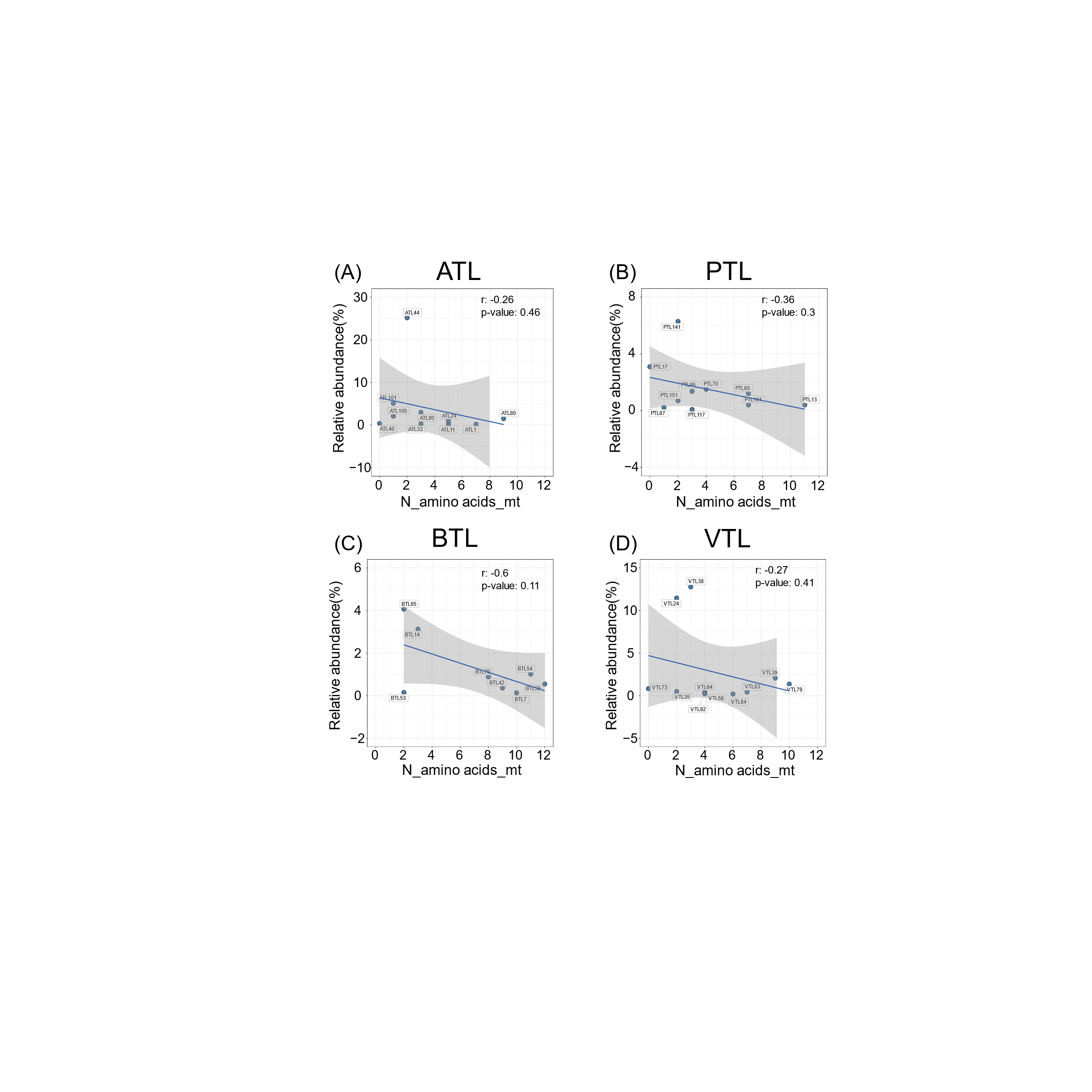


**Fig. S8.** The correlation between the abundance and the ability of amino acid synthesis of the MAGs belonging to SAOB at transcriptomic level. The ATL, PTL, BTL and VTL represent the thermophilic methanogenic chemostats supplemented with acetate, propionate, butyrate and isovalerate as sole carbon source, respectively. The “N_amino acids_mt” represents the number of amino acids of which the genes were actively transcribed in each MAG. The shaded area represents the 95% confidence region.


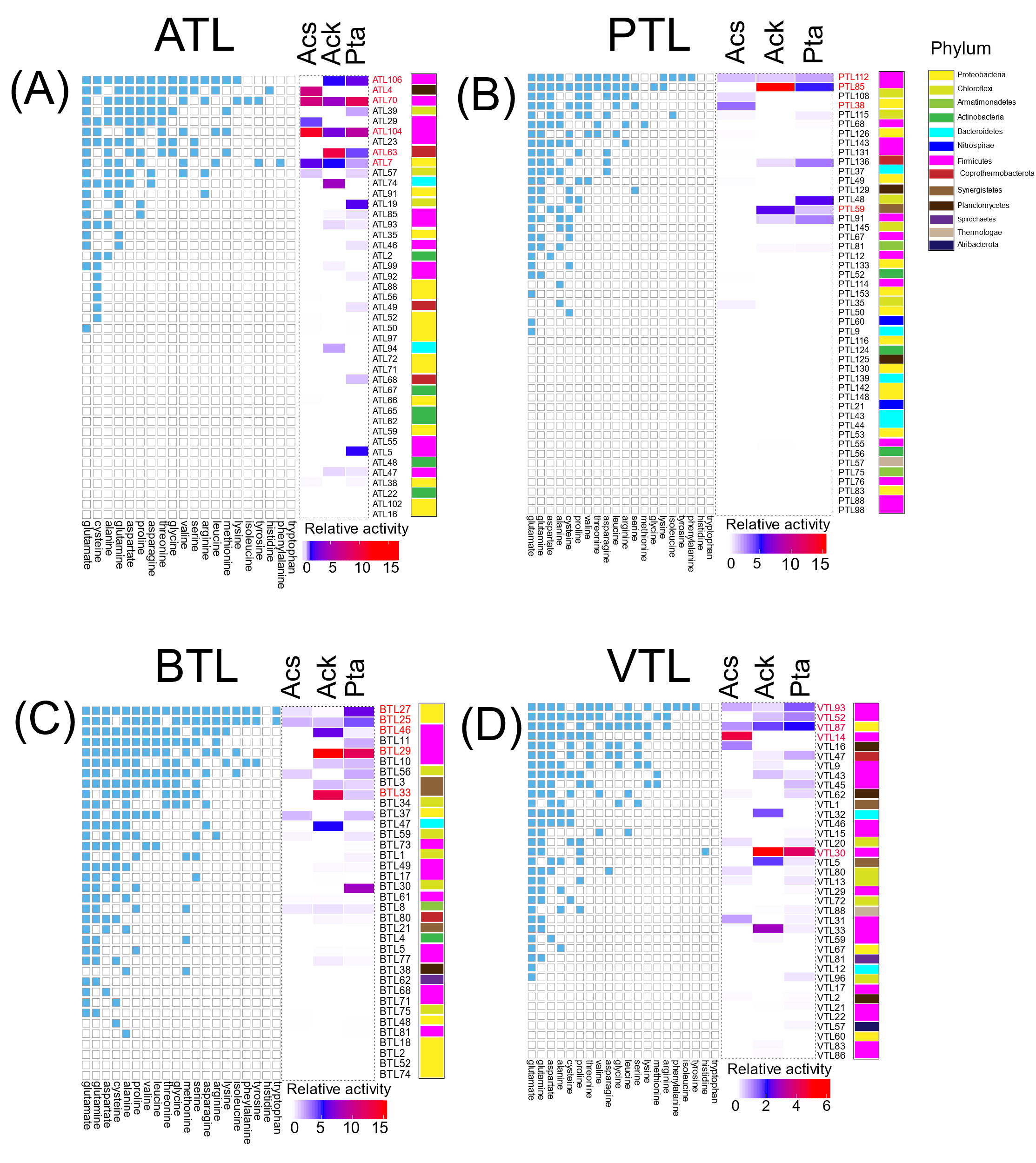


**Fig. S9.** The expression of genes encoding Acs, Ack and Pta in non-core functional bacterial MAGs. The ATL, PTL, BTL and VTL represent the thermophilic methanogenic chemostats supplemented with acetate, propionate, butyrate and isovalerate as sole carbon source, respectively. The landscape of amino acids (AAs) synthesis ability at the transcriptome level are shown on the left side. Each blue box denotes that the genes responsible for the synthesis of the corresponding AA were actively transcribed in the corresponding MAG. Shown on the right side are the relative activity (*RA_gene_*) of Acetyl-coenzyme A synthetase (Acs), Acetate kinase (Ack) and Phosphate acyltransferase (Pta) in non-core functional bacterial MAG, estimated as follow

${RA}_{gene}= {Activity}_{gene}*\frac{{MAG}_{i} reads}{\sum_{1}^{n} {MAG}_{i} reads}$

Here, *Activity_gene_* represents the RPKM-NM (see materials and methods) of each gene; *MAG_i_ reads* represents the number of reads per MAG at transcariptional level; *n* represents the number of all MAGs in each chemostat.

# References

Griffiths, R.I., Whiteley, A.S., O'Donnell, A.G., Bailey, M.J. (2000). Rapid method for coextraction of DNA and RNA from natural environments for analysis of ribosomal DNA- and rRNA-based microbial community composition. *Appl Environ Microbiol*. 66, 5488-5491. doi: 10.1128/AEM.66.12.5488-5491.2000

Anthony, M.B., Marc, L., Bjoern, U. (2014). Trimmomatic: a flexible trimmer for Illumina sequence data, Bioinformatics. 30, 2114–2120. doi: [10.1093/bioinformatics/btu170](https://doi.org/10.1093/bioinformatics/btu170)

Bankevich, A., Nurk, S., Antipov, D., Gurevich, A.A., Dvorkin, M., Kulikov, A.S., et al. (2012). SPAdes: a new genome assembly algorithm and its applications to single-cell sequencing. *J Comput Biol*. 19, 455-477. doi: 10.1089/cmb.2012.0021

Kang, D.D., Froula, J., Egan, R., Wang, Z. (2015). MetaBAT, an efficient tool for accurately reconstructing single genomes from complex microbial communities. *PeerJ*. 3, e1165. doi: 10.7717/peerj.1165

Parks, D.H., Imelfort, M., Skennerton, C.T., Hugenholtz, P., Tyson, G.W. (2015) CheckM: assessing the quality of microbial genomes recovered from isolates, single cells, and metagenomes. *Genome Res.* 25, 1043–1055. doi: [10.1101/gr.186072.114](https://doi.org/10.1101/gr.186072.114)
